# Label-free metabolic optical biomarkers track stem cell fate transition in real time

**DOI:** 10.1126/sciadv.adi6770

**Published:** 2024-05-08

**Authors:** Hao Zhou, Irene Li, Charles S. Bramlett, Bowen Wang, Jia Hao, Daniel P. Yen, Yuta Ando, Scott E. Fraser, Rong Lu, Keyue Shen

**Affiliations:** ^1^ Department of Biomedical Engineering, University of Southern California, Los Angeles, CA 90089, USA.; ^2^ Department of Stem Cell Biology and Regenerative Medicine, University of Southern California, Los Angeles, CA 90033, USA.; ^3^ Translational Imaging Center, University of Southern California, Los Angeles, CA 90089, USA.; ^4^ Molecular and Computational Biology, University of Southern California, Los Angeles, CA 90089, USA.; ^5^ Norris Comprehensive Cancer Center, University of Southern California, Los Angeles, CA 90033, USA.; ^6^ Department of Medicine, University of Southern California, Los Angeles, CA 90033, USA.; ^7^ USC Stem Cell, University of Southern California, Los Angeles, CA 90033, USA.

## Abstract

Tracking stem cell fate transition is crucial for understanding their development and optimizing biomanufacturing. Destructive single-cell methods provide a pseudotemporal landscape of stem cell differentiation but cannot monitor stem cell fate in real time. We established a metabolic optical metric using label-free fluorescence lifetime imaging microscopy (FLIM), feature extraction and machine learning–assisted analysis, for real-time cell fate tracking. From a library of 205 metabolic optical biomarker (MOB) features, we identified 56 associated with hematopoietic stem cell (HSC) differentiation. These features collectively describe HSC fate transition and detect its bifurcate lineage choice. We further derived a MOB score measuring the “metabolic stemness” of single cells and distinguishing their division patterns. This score reveals a distinct role of asymmetric division in rescuing stem cells with compromised metabolic stemness and a unique mechanism of PI3K inhibition in promoting ex vivo HSC maintenance. MOB profiling is a powerful tool for tracking stem cell fate transition and improving their biomanufacturing from a single-cell perspective.

## Teaser

Blood stem cell fates can be traced noninvasively by metabolic imaging-based biomarkers, shedding light on regenerative medicine.

## INTRODUCTION

The complex and dynamic fate transition of stem cells plays a crucial role in tissue/organ development (*1*), regeneration (*2*), and disease progression (*3*). The classic step-wise differentiation model regards stem cells and their offspring progenitors and mature cells as discrete, homogeneous populations, often defining them by specific combinations of surface markers (*4*). Yet, the intermediate status during stem cell fate transition and associated cellular reprogramming have been largely unknown. Meanwhile, considerable heterogeneity has been observed in the fate choices of different stem cell clones without conclusive mechanisms (*5*). At the single-cell level, stem cell fate is plastic and can be influenced by various factors and events, such as the interactions with niche microenvironment (*6*), epigenetic priming (*7*), and asymmetric division (*8*). Techniques capable of measuring the dynamic and heterogeneous stem cell fates thus hold the key to understanding the transitional states of stem cell differentiation, elucidating the precise factors regulating such processes and unlocking the full potential of stem cells for cellular therapies and regenerative medicine.

Lately, transcriptomic and proteomic data and pseudotime analysis indicate that cell fate transition happens in a continuum where stem cells gradually progress through the progenitor stages to become lineage-committed cells (*9*). However, such trajectory has been inferred from cell population data by densely sampling cells at different stages (*10*, *11*). These omics approaches also require destroying the cells and are thus impossible to determine the exact developmental trajectory of individual cells (*12*). To address such limitations, there is a growing interest in directly monitoring stem cell fate by methods that do not require destructive sampling. Live imaging allows observation of individual cells as they differentiate, providing more detailed insights into the transitional states and continuum. Nevertheless, conventional imaging modalities usually monitor stem cells through reporter genes or surface protein markers, which only provide limited information due to technical challenges to encode multiple reporters and/or a lack of functional meaning of the used markers (*8*). A comprehensive, noninvasive, function-based tool is needed to reveal the dynamic fate transition of stem cells in real time.

Cell metabolism is strongly associated with stem cell identity and heterogeneity (*13*) and adapts to environmental and epigenetic factors (*14*). It has been shown that glycolysis supports the quiescence and multipotency, while activated mitochondrial oxidative phosphorylation (OXPHOS) is required for the differentiation of hematopoietic stem cells (HSCs) (*15*), mesenchymal stem cells (*16*), and neural stem cells (*17*). The preference for anaerobic glycolysis over OXPHOS was further proposed as the “metabolic stemness” for HSCs (*15*). Moreover, cell metabolism changes dynamically and rapidly during stem cell fate transition (*8*). Therefore, analyzing metabolic states offers a unique opportunity for monitoring stem cell fate in real time.

Nicotinamide adenine dinucleotide (phosphate) [NAD(P)H] and flavin adenine dinucleotide (FAD) are two auto-fluorescent metabolic coenzymes involved in glycolysis and/or OXPHOS and play a critical role in stem cell functions (*18*). The autofluorescence of NAD(P)H and FAD can be detected in a label-free manner with fluorescence microscopy, including the fluorescence lifetime imaging microscopy (FLIM). FLIM measures the fluorescence intensity and lifetime (i.e., the characteristic time of fluorescence decay) of NAD(P)H and FAD, which depend largely on the coenzyme levels and their binding status with different metabolic enzymes (*19*). We have previously revealed a relationship between FLIM readouts and glycolysis/OXPHOS states in HSCs (*20*). In the present study, we used mouse HSCs as a model to build a framework that combines noninvasive metabolic imaging (i.e., FLIM), machine learning tools, and trending-based analysis to track stem cell fate transition in the continuum at the single-cell level.

## RESULTS

### Selected MOB features profile HSC differentiation continuum

**Fig. 1.**
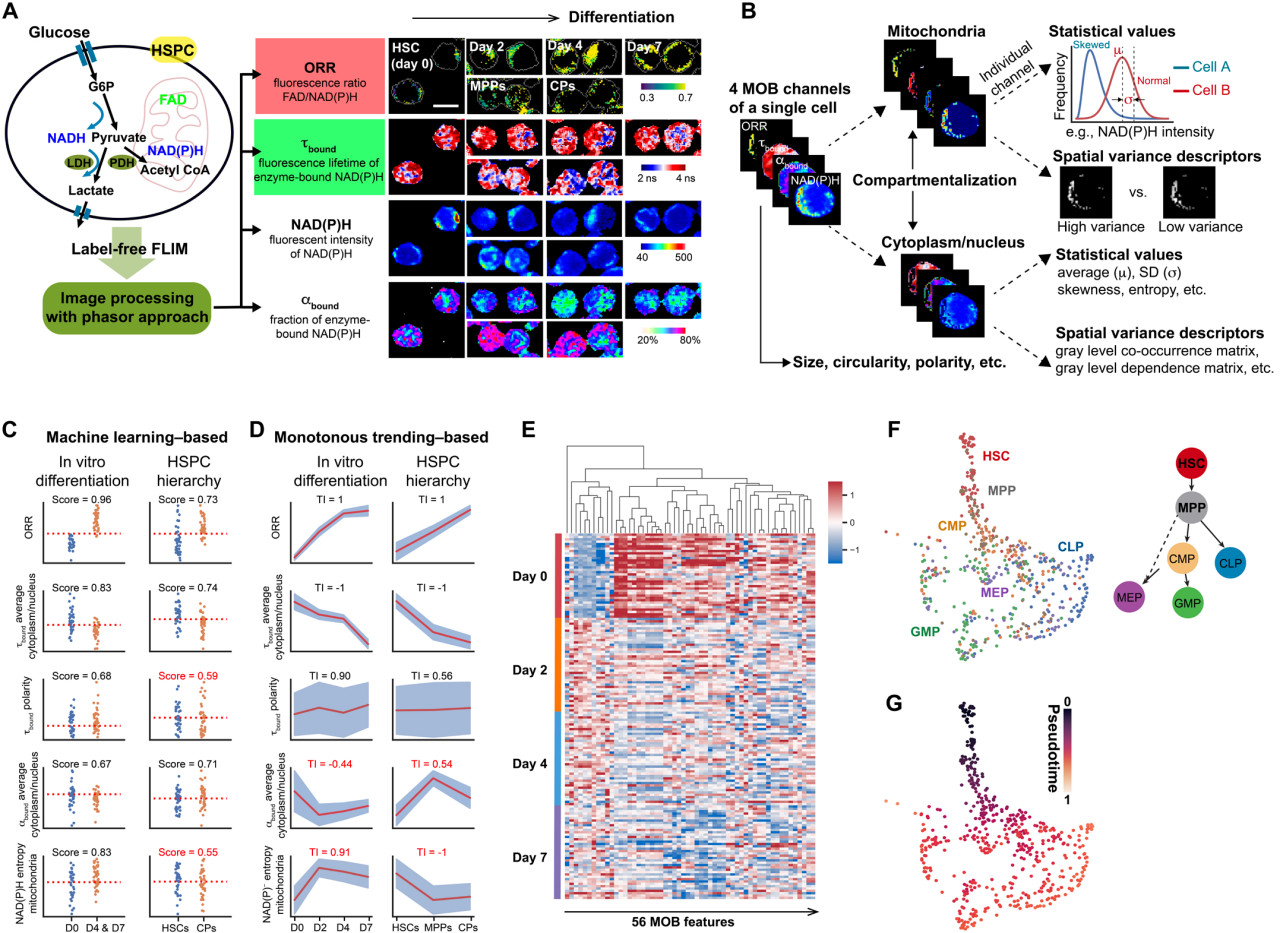
MOB features track the HSC differentiation continuum. (**A**) Schematic of FLIM profiling of the metabolic phenotypes of hematopoietic stem and progenitor cells (HSPCs) (left) and representative images of HSCs in in vitro differentiation and freshly isolated HSPCs along the differentiation hierarchy (right). ORR reflects cellular redox state and is sensitive to mitochondrial OXPHOS, and τ_bound_ is sensitive to lactate dehydrogenase activity in glycolysis. LDH, lactate dehydrogenase; PDH, pyruvate dehydrogenase; CoA, coenzyme A; MPPs, multipotent progenitors; CPs, common progenitors. Scale bar, 10 μm. (**B**) Schematic of single-cell mitochondria versus cytoplasm/nucleus segmentation and feature extraction from individual MOB channels. (**C**) Examples of MOB features examined by the machine learning–based selection. Red dashed lines indicate the decision boundaries in the logistic regression prediction models. (**D**) Examples of MOB features examined by the monotonous trending–based selection. Red lines connect the average of each time point/population and gray zones indicate the 95% confidence interval. *n* = 156 cells for HSC in vitro differentiation and 437 cells for HSPC hierarchy. Red texts highlight the cases where MOB features fail to pass the selection criteria. (**E**) Heatmap of 56 selected MOB features from HSC in vitro differentiation (*n* = 156 cells). (**F**) Illustration of HSPC hierarchy with myeloid and lymphoid lineages (right) and the trajectory interference using 56 MOB features (left, *n* = 548 cells). CMP, common myeloid progenitor; CLP, common lymphoid progenitor; GMP, granulocyte/macrophage progenitor; MEP, megakaryocyte/erythrocyte progenitor. (**G**) Pseudotime analysis of cells from HSPC hierarchy.

HSCs proliferate and differentiate in vivo or upon cytokine stimuli in vitro (fig. S1) (*21*), which offers two models of HSC differentiation. Using two-photon FLIM, we obtained high-resolution images of HSCs and the more differentiated hematopoietic progenitors from freshly isolated bone marrow (tables S1 and S2) or from extended in vitro HSC cultures. These images record the intensity and lifetime changes of the autofluorescence of NAD(P)H and FAD (*19*), during HSC differentiation. Four channels of metabolic optical biomarkers (MOBs) were derived from the raw images to represent the respective metabolic phenotypes, including fluorescence intensity of NAD(P)H, optical redox ratio [ORR, defined as FAD/NAD(P)H, associated with redox state and OXPHOS], fraction of NAD(P)H bound to enzymes (α_bound_), and fluorescence lifetime of enzyme-bound NAD(P)H (τ_bound_, associated with glycolysis) (Fig. 1A and fig. S2) (*20*). Next, we established a comprehensive library of 205 cellular and subcellular MOB-based features from every single cell (Fig. 1B and data S1), which include (i) morphological features (size, shape, etc.); (ii) signal strength, statistical distribution, and spatial variance of the MOBs in the cytoplasmic/nuclear and mitochondrial regions, respectively (figs. S3 and S4); and (iii) difference of the MOBs between the cytoplasmic/nuclear and mitochondrial regions (i.e., compartmentalization) (*22*). The defined cellular and subcellular features allow detailed profiling of HSC differentiation from high dimensions.

To identify MOB features that best describe HSC differentiation, we then established criteria through both machine learning and monotonous trending-based approaches (Fig. 1, C and D). In the machine learning route, we fitted a logistic regression model to each MOB feature to highlight and select those that can predict HSCs against the differentiated cells in both differentiation models (Fig. 1C). In the monotonous trending analysis route, we hypothesized that stemness-related MOB features should exhibit consistent up- or downward trends during HSC differentiation in both models (Fig. 1D). On the basis of these principles and assumptions, 56 MOB features were selected to represent the metabolic shift during HSC differentiation (Fig. 1E and fig. S5A). To validate whether these 56 MOB features can truly profile HSC differentiation, we collected different oligopotent progenitors downstream of HSCs in the hematopoietic lineage hierarchy, including the myeloid and lymphoid lineage progenitors (Fig. 1F, right). Intriguingly, our pseudotime analysis and trajectory inference (*23*) with the 56 MOB features revealed the continuum and bifurcate trajectories in HSC differentiation and lineage choices (Fig. 1, F and G, and fig. S5B), suggesting that the established optical metabolic signature captures the critical features during HSC fate decision.

### MOB score robustly quantifies HSC differentiation

**Fig. 2.**
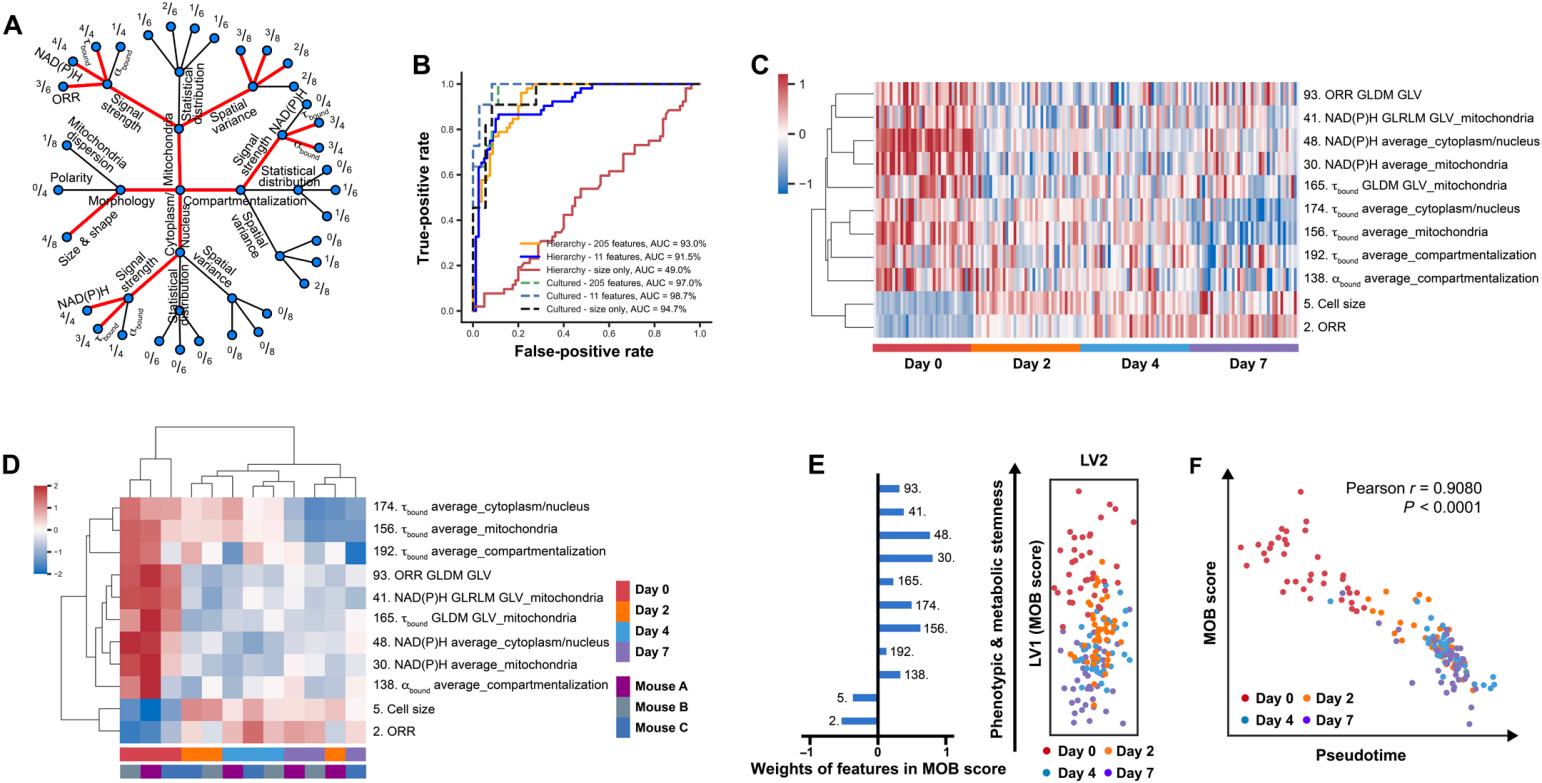
MOB score is a robust metric for HSC differentiation. (**A**) Knowledge graph visualizing the selection process of representative MOB features, which reflects the feature definition and the hierarchical relationship. Fractions on the nodes indicate the number of features that pass the selection process in both Fig. 1 (C and D). Highlighted edges indicate the subcategories that include the eventually selected MOB features. (**B**) ROC curve indicating the prediction accuracy by the SVM models using different features in HSC in vitro differentiation and HSPC hierarchy. AUC, area under the curve. (**C**) Heatmap of 11 representative MOB features from HSC in vitro differentiation. *n* = 156 cells. (**D**) Hierarchical clustering showed that MOB features can track HSC differentiation across independent experiments for in vitro differentiation. (**E**) Latent variable analysis of HSC in vitro differentiation and the weights of 11 representative MOB features. (**F**) Correlation between MOB score and pseudotime derived from 56 MOB features.

Although pseudotime analysis can resolve the position of each single cell in the differentiation trajectories by nonlinear clustering and path inference, it can only be achieved retrospectively with data collected from a large number of cells. We seek to establish a MOB feature–based metric to access the stemness of a single cell in real time. To achieve this goal, we first examined the phenotypes profiled by the 56 selected MOB features. We generated a knowledge graph to illustrate the biological meanings and relationships and found 11 highly independent features to represent the 56 features (Fig. 2, A to C; fig. S6A; and Materials and Methods). To demonstrate the predictive power of the representative features, we trained three support vector machine (SVM)–based classification models with (i) a single feature (cell size), (ii) the 11 representative features, and (iii) all the 205 features and evaluated their performance in predicting HSCs from freshly isolated progenitors or cultured HSCs at days 2 to 7 (Materials and Methods). The receiver operating characteristic (ROC) curves indicated that the SVM models trained with the 11 MOB features reached an accuracy similar to that with all 205 features or 56 selected features (Fig. 2B and fig. S6, B and C). A closer examination of the biological meanings of the 11 MOB features suggested a trend of increased cell size and a metabolic switch from anaerobic glycolysis to OXPHOS during HSC differentiation (Fig. 2C and table S3), consistent with the previous reports (*13*, *15*). Moreover, the NAD(P)H level, the spatial heterogeneity of mitochondrial metabolism [described as the variances of mitochondrial NAD(P)H intensity, ORR, and τ_bound_], and the metabolic distinction between the mitochondrial and cytoplasmic/nuclear regions (described as the α_bound_ and τ_bound_ compartmentalization) also decreased with differentiation (Fig. 2C and fig. S6A). We further found that the unsupervised clustering of the 11 MOB features accurately predicted the stemness of cells in freshly isolated populations (fig. S6D) and extended cultures (Fig. 2D) in three independent studies with different donor mice, respectively, confirming the robustness of the 11 features in tracking HSC differentiation.

To quantitatively describe the stemness of a single cell in the HSC differentiation continuum, we used a latent variable model to combine the 11 representative MOB features into a single, numeric score (i.e., the MOB score). This model is linear and can explain explicitly the contribution of individual MOB features to the stemness (Fig. 2E and fig. S6, E and F). We benchmarked the MOB score with the state-of-art pseudotime analysis algorithm (*23*). The resultant nonlinear “pseudotime” in differentiation was inversely correlated with the linear MOB score (i.e., metabolic stemness) at the single-cell level (Fig. 2F), confirming the ability of the MOB score in tracking the HSC differentiation status.

### MOB score detects metabolic inheritance and symmetric/asymmetric division

**Fig. 3.**
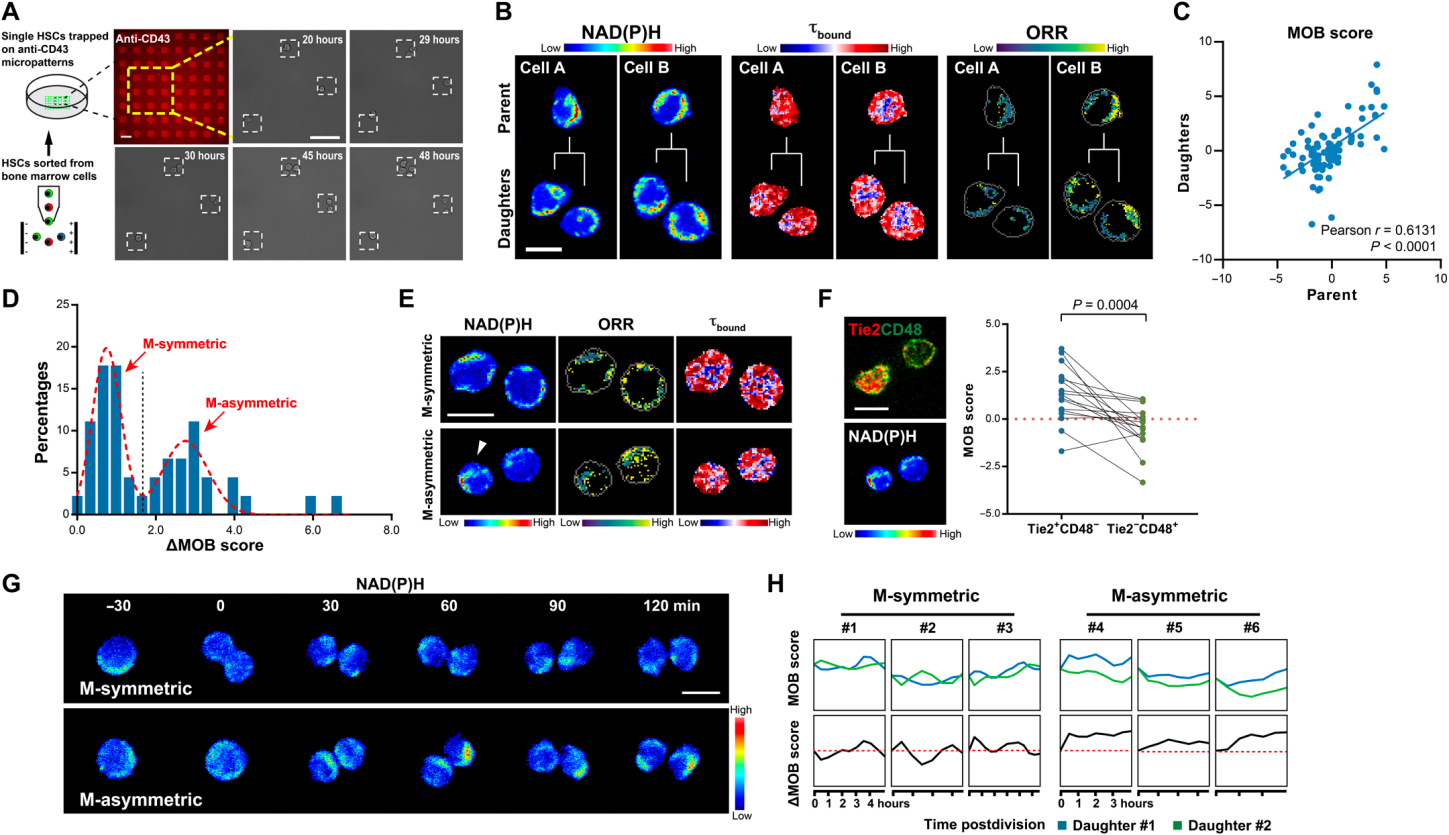
MOB score identifies metabolic inheritance and asymmetry in HSC division. (**A**) Illustration and representative images of single HSCs trapped on the micropattern array and tracked during division. Scale bar, 50 μm. (**B**) Representative MOB images of individual HSCs and their daughter cells. Scale bar, 10 μm. (**C**) Correlation of MOB scores between parent HSC and PDCs. *n* = 44 divisions. (**D**) Histogram of ΔMOB scores in PDCs from the first division of HSCs in vitro. *n* = 45 cell pairs. (**E**) Representative MOB images of PDCs from metabolically symmetric (M-symmetric) and asymmetric (M-asymmetric) divisions. Arrows indicate the stem-like daughter cell from an M-asymmetric division. Scale bar, 10 μm. (**F**) Representative images of phenotypically identified PDCs from asymmetric division and the corresponding MOB images (left) and correlation between Tie2CD48 phenotypes and MOB scores in PDCs (right). Scale bar, 10 μm. Red dashed line: threshold between stem and differentiated cells derived from maximum likelihood estimation. *n* = 17 cell pairs. *P* values: paired *t* test. (**G**) NAD(P)H time-lapse images from metabolically symmetric and asymmetric divisions. Scale bar, 10 μm. (**H**) Dynamic changes in MOB scores in PDCs after division.

The observed gradual decline of stemness in the extended HSC culture (Fig. 2, E and F) highlights a continuum in HSC fate transition during differentiation at the population level. We next aim to track how such continuum appears at the single-cell level and the role of cell division in this process. MOB scoring allows for a direct, temporal comparison of the metabolic stemness of daughter cells and their parent HSCs at the single-cell level. Since HSCs are suspension cells, we used a micropatterning approach to constrain them for continuous observation over a 48-hour period of culture time (Fig. 3A and fig. S7A). MOB scores were calculated in individual HSCs and their paired daughter cells (PDCs) before and after the division, respectively (Fig. 3B). HSCs are mostly quiescent in vivo and were reported to be metabolically activated upon in vitro culture (*24*). We noticed an increase in ORR on day 1, suggesting an up-regulation in OXPHOS activity. Furthermore, the MOB score significantly decreased on day 1, followed by a partial recovery postdivision (fig. S7, B and C). This trend is consistent with the metabolic transition HSCs undergo from the activation state to a postdivision quiescent state (*24*). We observed a notable similarity in MOB features and a significant correlation in the MOB scores between the parent HSCs and their corresponding offspring (Fig. 3, B and C), which suggests an inheritance of the metabolic phenotype at the single HSC level. Such inheritance seems broadly applicable in different differentiation stages, as the correlation in MOB scores between parent and daughter cells was seen in all three hematopoietic stem and progenitor cell (HSPC) populations examined in this study (fig. S7D). Our results here thus indicate that metabolic stemness is a relatively stable characteristic in the HSPCs, with an “inertia” from parent to daughter cells during cell division. Such observation also agrees with the reported epigenetic memory in stem cell differentiation at the clonal level (*7*).

Besides the overall inheritance, some PDCs from the same parent cells also displayed relatively divergent metabolic stemness (Fig. 3C). As daughter cells may assume similar or different fates through symmetric or asymmetric divisions (*25*), we next examined the division patterns of individual HSCs using the MOB score. Notably, we observed two major peaks in the histogram of the MOB score differences in PDCs (ΔMOB scores), indicating the existence of the two distinct division patterns (Fig. 3D). Using a double Gaussian mixture model, we determined the threshold value that separates the two peaks to define the metabolically symmetric (M-symmetric) and asymmetric (M-asymmetric) divisions (Fig. 3, D and E). To assess whether such metabolic asymmetry reflects distinct fates of the two daughter cells, we immuno-stained the FLIM-measured PDCs for Tie2 and CD48, a set of binary surface markers previously used to identify asymmetric division in HSCs (*26*). The more stem-like daughter cells (Tie2^+^CD48^−^) had significantly higher MOB scores than their committed siblings (Tie2^−^CD48^+^) (Fig. 3F).

The ability to distinguish the subtle difference between PDCs makes the MOB features and score appealing metrics for tracking stem cell division in real time. To demonstrate such feasibility, we monitored the single dividing HSCs with time-lapse imaging at 30-min intervals. We made an unexpected discovery that the NAD(P)H intensities and MOB scores of the sibling cells were similar to each other immediately upon the cytoplasmic division (within ~60 min); specifically in M-asymmetric PDCs, their metabolic profiles began to diverge thereafter (Fig. 3, G and H). Our results suggest that the observed metabolic asymmetry is regulated by some upstream determinants rather than the differential inheritance of the metabolic coenzymes [e.g., NAD(P)H].

### Daughter cells have divergent fate decisions under different division patterns

**Fig. 4.**
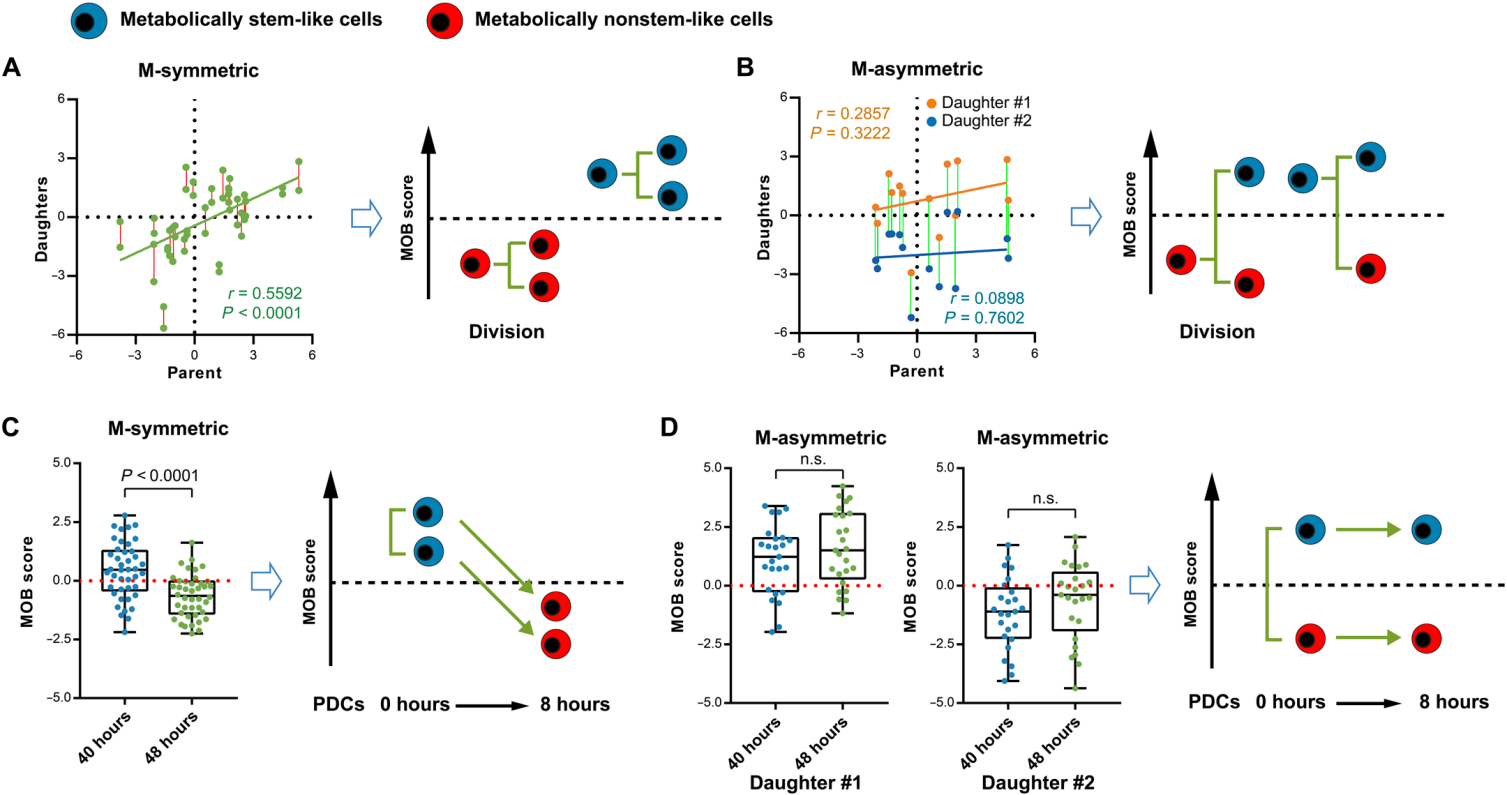
MOB score tracks divergent fate decisions of daughter cells from different division patterns. (**A** and **B**) Inheritance and variation of metabolic phenotypes under different division patterns. Red or green lines connect the PDCs from the same parents. *n* = 39 divisions. (**C** and **D**) Maintenance/loss of metabolic stemness of PDCs from different division patterns. *n* = 67 cell pairs. *P* values: Mann-Whitney test.

Another unexpected feature of the M-asymmetric division is the parent-daughter relationship in MOB scores. As noted earlier, HSCs with higher MOB scores generally give rise to stem-like daughter cells (Fig. 3C). Such correlation still existed and was significant for the M-symmetrically divided pairs (Fig. 4A). However, there were poor correlations between the parent and either of the two daughter cells in the M-asymmetrically divided HSCs (Fig. 4B). This was largely because some HSCs with lower (or higher) MOB scores regained (or lost) metabolic stemness in one of their daughter cells through the M-asymmetric division (Fig. 4B). Moreover, the M-asymmetric division seems to be a more reserved feature of stem cells, as we found that HSCs were more likely to divide asymmetrically than the progenitors (fig. S8).

Another key question is, which division pattern is primarily responsible for stem cell loss during in vitro culture? We tracked the MOB scores of PDCs after division for additional 8 hours. Upon division, over half of the daughter cells from M-symmetric division had MOB scores above the threshold line of “metabolically-stem,” suggesting their initial stem-like status; in contrast, daughter cells from M-asymmetric division showed divergent metabolic stemness. However, 8 hours later, the MOB scores of the PDCs from M-symmetric division had dropped below the threshold line to become similar to the committed daughter cells from M-asymmetric division (Fig. 4C); in contrast, those more stem-like daughter cells from M-asymmetric division remained stem-like (Fig. 4D). Our results suggest that without further intervention, M-symmetric division leads to a quicker loss of the HSC population, while M-asymmetric division better preserves the stem cell pool under the normal cytokine culture condition. These results thus suggest a unique role of asymmetric division in maintaining the HSC population.

### MOB score–based analysis finds culture conditions promoting HSC maintenance

**Fig. 5.**
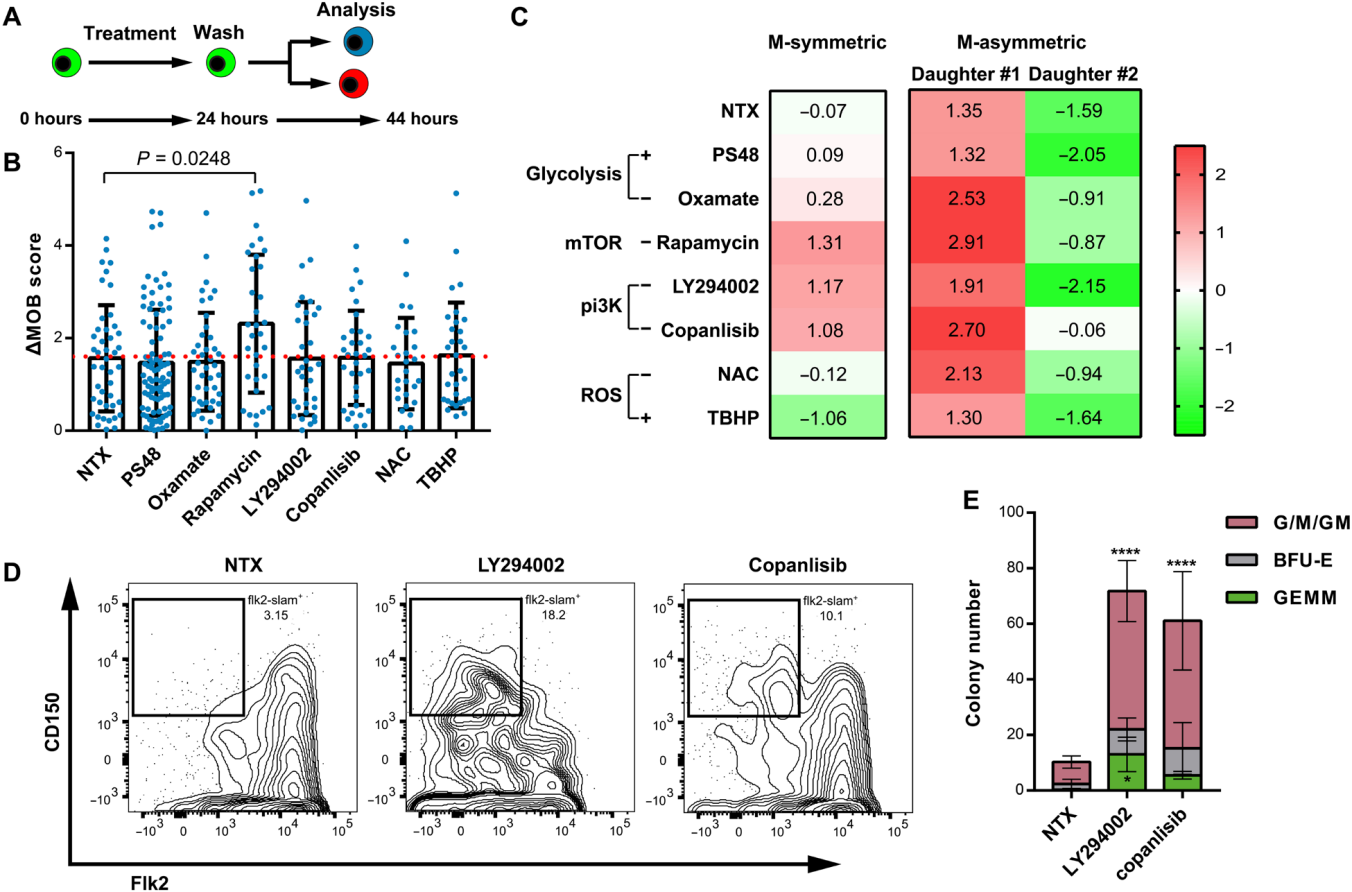
MOB score identifies conditions that promote HSPC maintenance/expansion. (**A**) Experimental design studying the influence of metabolic intervention on HSC division pattern. (**B**) PDC metabolic asymmetry induced by metabolic intervention on parent HSCs. *n* = 333 cell pairs. *P* values: one-way analysis of variance (ANOVA). (**C**) Average MOB score of PDCs under different division patterns after metabolic intervention on parent HSCs. (**D**) Flow cytometry analysis of the CD150^+^Flk2^−^ fraction of KLS (cKit^+^Lin-Sca^+^) cells after 1-week in vitro culture of freshly isolated HSCs. (**E**) Colony-forming ability per 1000 cultured cells under pan-PI3K inhibition. G/M/GM, CFU–granulocyte/macrophage/granulocyte and macrophage; BFU-E, burst-forming unit–erythroid; GEMM: CFU–granulocyte, erythrocyte, macrophage, megakaryocyte. *n* = 3 biological replicates. *P* values: one-way ANOVA. *P < 0.05; ****P < 0.0001. NAC, N-acetylcysteine; NTX, no treatment.

Our observation suggests an opportunity to improve stem cell biomanufacturing by modulating the metabolism of parent stem cells. We shortlisted seven candidate drugs that have been reported to target metabolic pathways (table S4). We treated HSCs in the micropatterned culture with these drugs for 24 hours, before washing the drugs out and measuring the ΔMOB scores in PDCs at 44 hours (Fig. 5A). A comparison of the ΔMOB scores between the treated and the nontreated groups showed that only rapamycin promoted M-asymmetric division (Fig. 5B). Notably, M-asymmetric division stably generated two divergent daughter cells, even under the oxidative stress induced by tert-Butyl hydroperoxide. Other than rapamycin, which has been shown to promote HSC expansion (*27*), pan-PI3K inhibitors LY294002 and copanlisib also promoted the MOB scores of the PDCs from M-symmetric division (Fig. 5C), suggesting that they may prevent or slow the loss of stem cells. To test this hypothesis, we cultured freshly isolated HSCs for 1 week under the treatment of the two PI3K inhibitors, respectively. Flow cytometric analysis showed that the pan-PI3K inhibition markedly increased the fraction of Flk2-CD150^+^ KLS (cKit^+^Lin-Sca1^+^) cells (presumably phenotypic HSCs) (Fig. 5D). We then performed colony-forming unit (CFU) assay to confirm the multilineage differentiation capacity of these cultured cells. Our results showed that the LY294002 and copanlisib-treated cells formed more colonies as well as the most primitive granulocyte/erythrocyte/monocyte/megakaryocyte (GEMM) colonies (Fig. 5E). These results suggest that the MOB score–based division pattern analysis may be integrated in screening workflow to optimize stem cell biomanufacturing.

## DISCUSSION

Stem cells are heterogeneous and have plasticity in fate decisions. Studying their fate transition can help us understand developmental biology and provide guidance for the ex vivo maintenance or expansion of stem cells. While metabolism is a promising indicator of single stem cell fate transition, previous studies on stem cell metabolism have been mostly performed with destructive methods or end-point measurements like mass spectrometry (*28*) or Seahorse Assays (*29*), which also require a large number of cells. Single-cell metabolic staining using invasive dyes [e.g., 2-(7-nitro-2,1,3-benzoxadiazol-4-yl)-d-glucosamine for glycolysis and tetramethylrhodamine, methyl ester (TMRM) for mitochondrial activity] (*13*, *30*), on the other hand, may interrupt the native metabolism through the dyes or special buffers (*31*). These dyes also involve different loading procedures that may interfere with each other, thus limiting the possibility of tracking multiple metabolic pathways in the same cell. In contrast, the FLIM approach used here is advantageous by providing a noninvasive, real-time solution to simultaneously track multiple metabolic indicators/pathways at the single-cell level.

When using FLIM in specific biological contexts, it is important to note the potential presence of other sources of autofluorescence within cells (*19*). To study hematopoietic cells with FLIM, we have undertaken several measures to ensure that the observed signals are predominantly from NAD(P)H and FAD (*20*). This includes biochemical assays correlating fluorescence signals with actual NAD(P)H levels in different hematopoietic cell types, pharmacological manipulations affecting NAD(P)H-cycling enzymes with subsequent measurement of NAD(P)H fluorescence changes, and the close monitoring of fluorescence lifetimes in phasor plots to distinguish NAD(P)H and FAD from other autofluorescent compounds. These steps collectively confirm that the observed trends reflect changes in NAD(P)H and FAD metabolism. While we cannot completely rule out minor contributions from other autofluorescent species, the evidence suggests that they do not substantially affect the overall interpretation of our data.

Photon budget is a crucial consideration in live-cell NAD(P)H FLIM. Ideally, hundreds to thousands of photons per pixel are needed for reliable biexponential fitting in fluorescence lifetime analysis (*32*). However, such high photon counts often lead to phototoxicity, a well-documented challenge in autofluorescence-based live-cell FLIM studies (*33*). In practice, we have observed an abnormal shift in the FLIM profile of HSCs (indicating cellular stresses caused by imaging) within 30 min under the excitation laser power just 20 to 30% higher than the power setting used in this study. Consequently, we adopted a more conservative photon budget during the continuous live-cell imaging. This photon budget is in line with the current practices of NAD(P)H FLIM imaging in live cells and reflects a practical compromise to mitigate the risk of phototoxicity while securing meaningful data (*34*, *35*). To address the curve-fitting challenges associated with low photon counts, we have taken the strategies of fit-free phasor transformation, median filtering, and fixing τ_free_ in phasor plot–based analysis to calculate τ_bound_ and α_bound_ (see Materials and Methods), as our previous data showed that τ_free_ has no significant difference between various hematopoietic cell types (*20*). Moreover, the combinatorial analysis performed in this study may also reduce the influence of noises from individual FLIM parameters and improve the robustness of the results, a noise-reducing strategy taken by other high-dimensional dataset analyses (*36*). There is a high consistency between MOB scores derived from the original versus the 2 × 2 binned FLIM images (fig. S9), the latter of which effectively quadruples the photon count per pixel.

Previous studies have explored the pixel-level information from autofluorescence imaging, using intensity patterns for characterizing mitochondrial dynamics (*37*), correlating NAD(P)H lifetime with intracellular pH (*38*), and demonstrating FLIM signal heterogeneity within individual samples or cells (*19*, *35*). We also noticed that different subcellular regions, such as mitochondria and cytoplasm/nucleus, have distinct signal patterns including NAD(P)H intensity and lifetime. It was reported that NAD(P)H is compartmentalized in the mitochondria and cytoplasm of mammalian cells (*39*, *40*). We thus quantified the mitochondrial and cytoplasmic/nuclear signals separately as well as the degree of compartmentalization for every single cell. Moreover, it was previously shown that the health and functionality of individual mitochondria are associated with their fission/fusion dynamics and dispersity (*24*), which can be reflected by their metabolic heterogeneity (*41*). We thus quantified the statistical distribution and spatial variance of FLIM signals in mitochondrial regions, which profile the metabolic heterogeneity and quality of individual organelles. By including these spatial and statistical aspects, we defined a comprehensive library containing 205 MOB features to describe cellular metabolism from high dimensions.

We developed a machine learning–assisted workflow to identify the 56 features that vary consistently along HSC differentiation and further selected the 11 independent, representative features. During this process, the monotonous trend along HSC differentiation was adopted as a criterion for feature selection. It is important to note that stem cell dynamics can exhibit a range of patterns, not all of which are linear or monotonous. In our study, the focus on monotonous trends was a strategic choice aiming at establishing a linear model for its simplicity and adaptability. Such a model facilitates applications in various contexts (e.g., cell division) without the need for complex, nonlinear trajectory analysis. Moreover, from a technical standpoint, selecting MOB features exhibiting monotonous trends simplified the comparative analysis across different differentiation models, both in vivo and in vitro. To establish the linear model, we used the Robust Scaler to normalize each feature and a latent variable model to determine the weights. To further expand its utility across different biological systems, an adaptive feature-weighting algorithm may be integrated into the model. The weight of each feature can be assigned based on its statistical behavior across different samples (e.g., variance) and relevance to the target events (e.g., differentiation) across datasets. By doing so, features with consistent and significant patterns across datasets would be given higher weights, thus improving the robustness and applicability of the MOB score. It will enhance the generalizability and thus the utility and appeal of the MOB score system for broader interest in the field.

The selected MOB features contain information from metabolic pathways involved in HSC differentiation. For example, we and others have shown that τ_bound_ is sensitive to lactate dehydrogenase activity in glycolysis (*20*, *42*), while ORR is sensitive to OXPHOS (*43*). In this study, we observed a steady increase in ORR and decrease in τ_bound_ during HSC culture and along the HSPC hierarchy (Fig. 1D, top two rows), which is consistent with the reported metabolic rewiring from glycolysis to OXPHOS during HSC differentiation (*15*). Notably, τ_bound_ and ORR can also be influenced by other cellular and molecular activities, such as the interactions of NADH with different protein complexes. Thus, rather than definitive markers for glycolysis and OXPHOS, they should be used as informative indicators of these metabolic pathways. Despite these limitations, they remain valuable in providing possible metabolic clues due to a shortage of noninvasive tools for probing metabolism in single living cells. Cell size also increased markedly during in vitro HSC differentiation, likely due to the fast proliferation under cytokine stimulation. There are limited reports suggesting that cell size is a marker of HSC differentiation, while recently a machine learning–based analysis of differentiating hematopoietic progenitors found that cell size contributed to the prediction of lineage choices (*44*). However, cell size alone cannot reliably track HSC differentiation in the in vivo HSPC hierarchy (Fig. 2B). The 11 selected MOB features also include three spatial variance descriptors for mitochondria, indicating that the metabolism in mitochondrial regions within individual cells becomes more uniform during HSC differentiation. This result also suggests higher diversity in the quality and activity of individual mitochondria in HSCs compared to the differentiated cells, possibly as a result of more frequent mitochondrial fission and recycling of damaged mitochondria in HSCs (*24*). This is a conserved mitochondrial clearance mechanism in different adult stem cell types (*45*, *46*). Notably, a decrease in NAD(P)H level was observed during HSC differentiation, consistent with the reported role of NAD metabolism in promoting mitochondrial clearance in stem cells (*46*, *47*).

With the selected MOB features, we profiled the trajectory of HSC differentiation. Although the MOB features were selected only on the basis of the trend of differentiation, we observed the bifurcate trajectory of HSC differentiation in trajectory inference (Fig. 1F). This is consistent with the previous report that metabolism is a critical regulator of HSC lineage choice (*48*). However, the conventional pseudotime analysis of transcriptomic or proteomic data and mapping single cells to the established landscape require the destruction the cells (*49*–*52*). Our data showed that the subtle differences in the status of single cells can be noninvasively observed and mapped onto the differentiation trajectory, thus enabling cell fate tracking in real time and under dynamic scenarios.

Cell division is a critical checkpoint of stem cell fate decisions. The MOB score enabled us to measure the asymmetry in PDCs. To support the notion of metabolic differences between PDCs, we have also used dye staining methods using TMRM (which reports mitochondrial membrane polarization that correlates with OXPHOS level) (*8*, *13*) and 6NBDG (which reports glucose uptake that correlates with glycolytic level) (*53*). We indeed observed correlations between the MOB scores of PDCs and their 6NBDG (positive correlation) and TMRM (negative correlation) levels, respectively (fig. S10). Such asymmetry was associated with different fate markers determined with the immunostaining (*13*, *15*). A number of studies have underscored the role of OXPHOS in characterizing HSC fate (*13*, *15*, *54*), with increasing evidence pointing to its significance in indicating asymmetric cell division (*8*, *46*). We found that PDCs have similar metabolism immediately after division even in the M-asymmetric ones, and the asymmetry did not appear until about an hour later under our time interval settings (Fig. 3G). It was reported that PDCs have similar transcriptomes after cell division (*55*). In T lymphocytes, which can also commit asymmetric cell division, the expression of a metabolic regulator, c-Myc, becomes divergent in daughter cells only after the cell division (*56*). Our results are consistent with these observations and suggest that cell metabolism may act as an effector function downstream of some differentially regulated or inherited fate determinants (e.g., proteins, epigenetic modifications, cellular organelles, or extrinsic cues) (*57*–*60*). The timescale for the emergence of metabolic asymmetry in the PDCs after division was much shorter than what was reported with protein markers (*8*). This highlights the real-time ability of the MOBs in tracking rapid response and its potential in identifying new fate determinants or studying functional dynamics of such regulators in asymmetric division (*61*).

We have further shown that daughter cells from different division patterns have divergent abilities in obtaining/maintaining metabolic stemness. Unexpectedly, HSCs with low MOB scores (i.e., with rewired metabolism from glycolysis to OXHPOS during in vitro culture) can restore metabolic stemness through M-asymmetric division (Fig. 4B). Asymmetric division was believed as a clearance mechanism to maintain the potency of HSCs (*26*, *46*). By tracking cell fate after division, we found that the daughter cells from M-symmetric division quickly lose their metabolic stemness over time, while the stem-like daughter cells from M-asymmetric division can maintain their stemness (Fig. 4, C and D). Cancer stem-like cells have also been reported to maintain their stemness through asymmetric division, while their daughter cells from symmetric division become differentiated (*59*). These observations suggest symmetric division as a major cause of the loss of stem cell population during in vitro culture.

The ability to track single HSC fate transition offers a unique opportunity to improve HSC ex vivo expansion by regulating cell behaviors from a single-cell perspective. We revealed previously unknown role of two PI3K inhibitors in HSC symmetric division that can promote HSC maintenance. Recently, it was reported that a PI3K activator can be used as an alternative to stem cell factor (SCF) in human HSPC expansion (*62*). The activation of PI 3-kinase (PI3K)–AKT–mammalian target of rapamycin (mTOR) pathway, downstream of the SCF receptor cKit, is necessary for HSC cell cycle entry and proliferation. On the other hand, however, the overactivation of PI3K-AKT-mTOR pathway drives HSC differentiation and exhaustion (*63*). Collectively, these studies suggest that fine-tuning PI3K is critical for HSC maintenance. While the previous studies focus on the molecular mechanisms of PI3K-AKT-mTOR pathway in regulating mitochondrial respiration and reactive oxygen species production (*63*, *64*), our results from the single-cell perspective suggest that small-molecule inhibition of PI3K can rescue the metabolic stemness of daughter cells from symmetric division, which would otherwise decline.

We expect the MOB metric to provide a versatile platform and analytical framework that can promote HSC biomanufacturing. The conventional Seahorse Assays typically require a minimum of 10,000 HSCs per condition (*65*) and only provide the bulk average metabolic measurement. This presents logistical constraints on how many culture conditions can be realistically tested, especially considering the typical yield of 10,000 to 20,000 HSCs per adult mouse. In contrast, we can capture the metabolic profiles of approximately 200 to 400 densely loaded cells per condition in a single image with vast details of information on HSC behaviors and fate choices, although the throughput of the division pattern analysis is still limited under the current settings of micropatterning and low-density seeding. Yet, considering the rapid development of low-cost microtechnologies and emergence of automated single-cell level imaging-based drug screening platforms on the market, we foresee that our platform can be further scaled up and automated for screening optimal culture conditions in biomanufacturing, with the advantage of extremely low consumption of precious samples such as HSCs and other rare, primary stem cells.

Furthermore, the utility of our approach can be extended beyond in vitro cell cultures. For example, it may be used for in vivo analysis of single HSC divisions by leveraging the exceptional deep tissue penetration of the two-photon imaging systems (*66*). Such feasibility is supported by a number of two-photon microscopy-based intravital imaging studies of HSCs in the bone marrow of skull (*67*, *68*). In addition, we can combine our approach with a Hoxb5 mouse model, where HSCs can be readily identified with mCherry expression (*69*), thus allowing for tracking and studying the differentiation or division behaviors of HSCs in vivo using our label-free techniques. Overall, our MOB-based metric and analytical framework can potentially be applied to many other cell types or translated to other scenarios where dynamic cell fate transition needs to be closely monitored. This includes areas such as monitoring muscle stem cells during the repair of muscle damage, analyzing division patterns in circulating tumor cells, aiding in the diagnosis of leukemia, and guiding the process of directed cell reprogramming (*61*, *70*).

## MATERIALS AND METHODS

### Fluorescence-activated cell sorting

C57BL/6 mice were purchased from the Jackson Laboratory and bred at the Research Animal Facility of the University of Southern California. Animal procedures were approved by the Institutional Animal Care and Use Committee of the University of Southern California. Bone marrow cells were extracted from the crushed bones of 2- to 6-month-old C57BL/6 mice of mixed gender and then enriched using anti-cKit magnetic beads (Miltenyi) and immunostained for HSCs, multipotent progenitors (MPPs), and common progenitors (CPs) following our previously established protocol (tables S1 and S2) (*71*). Fluorescence-activated cell sorting (FACS) was carried out on a BD SORP FACSAria cell sorter at 4°C.

### Single-cell RNA sequencing data analysis

Single-cell RNA sequencing data for freshly isolated (GSE81682) and in vitro–cultured (GSM4185642) HSPCs were retrieved from previous publications (*5*, *49*). Data processing was carried out using the ScanPy package in Python 3.9 (*23*). Data was normalized, log-transformed, and rescaled to prepare for downstream analysis. Principal components analysis and uniform manifold approximation and projection were carried out for dimensionality reduction and cellular heterogeneity exploration. Cells were clustered using the Louvain method, and diffusion pseudotime analysis was conducted to infer differentiation trajectories. OXPHOS activity for each single cell was evaluated by scoring the mouse OXPHOS hallmark genes (*72*). The score is the average expression of the OPXHOS hallmark genes subtracted by the average expression of 50 randomly sampled genes from all genes.

### Single HSC micropatterning

Customized petridishes with #1 glass (0.15 mm in thickness) bottom were cleaned with detergent (7×, MP Biomedicals), rinsed, and dried before use. A photomask with micropatterns of 30 μm by 30 μm squares with 30-μm edge-to-edge spacing was designed with AutoCAD (Autodesk Inc.) and printed (International Phototool Company LLC). The photomask was used in photolithography with 50-μm SU-8 photoresist (MicroChem) on 7.62-cm silicon wafers to create molds for soft lithography. Sylgard 184 polydimethylsiloxane (Dow Corning) was fully mixed at 10:1 base-to-curing reagent ratio, poured onto mold, and baked at 80°C overnight. Stamps were then cut and peeled from the mold and coated with anti-CD43 (eBioR2/60, eBioscience) diluted at 25 μg/ml in Dulbecco’s phosphate-buffered saline (PBS; VWR Life Science). After 1 hour, the stamps were rinsed with 0.05% Tween 20 (Sigma-Aldrich) in PBS, PBS, and deionized water and dried with compressed air. Micropatterns were then transferred from stamps to the prepared petridishes with #1 glass bottom by microcontact printing. Freshly isolated HSCs were seeded at a density of 20 cells/mm^2^, so that the majority of the micropatterns accommodated 0 or only 1 cell.

### Fluorescence lifetime imaging

FACS-sorted cells were washed and resuspended in StemSpan SFEM medium (STEMCELL Technologies) supplemented with SCF (50 ng/ml) and thrombopoietin (50 ng/ml; PeproTech) (i.e., standard medium). For the comparison of freshly isolated HSC and progenitors, cells were resuspended at ~10^6^/ml, seeded in a 1536-well plate (Corning), and incubated for 1 hour before imaging. For the HSC in vitro culture and tracking, cells were resuspended at ~5 × 10^4^/ml and seeded in a 96-well plate (Corning). Half-medium was changed every 3 days. For the single HSC division study, cells were resuspended at ~1.5 × 10^4^/ml and seeded in a customized #1 glass-bottom petridish micropatterned with anti-CD43 (eBioR2/60, eBioscience) (*73*). Fluorescence lifetime images were acquired with a Leica STELLARIS 8 Fast Lifetime Contrast (FALCON) (previously referred to as Leica SP8 FALCON) inverted microscope with a live-cell workstation (37°C, 5% CO_2_) (Tokai Hit, Japan) except otherwise indicated. The microscope system uses time-correlated single photon counting for lifetime measurement, and the phasor FLIM distributions are derived from a Fourier transform (*74*) as data are acquired. The correction of phasor locations for the instrument response function is integral to the system’s design and functionality, and Coumarin 6 dye is regularly used to validate if it works as expected before image acquisition. This dye, known for its well-characterized fluorescence lifetime, serves as a standard to verify that the system accurately reflects its expected 2.5-ns lifetime at the edge of the semicircle in phasor plots. Intracellular NAD(P)H and FAD were excited at 740 nm in the two-photon mode; the emission wavelength/optical filter was 460/80 nm for NAD(P)H and 540/50 nm for FAD. The source laser power was rated at 1.8 W and was carefully controlled at 0.4 mW (for live-cell tracking) or 0.5 mW (for one-time imaging) at the exit aperture of the lens, to minimize the risk of photobleaching or cell damage during repeated imaging. The power level was directly measured with a photo-power meter from Thorlabs Inc. FLIM images were acquired at the pixel size of 0.18 μm and pixel dwell time of 15.38 μs, with 8× line repetitions.

### Calculation of ORR

The Leica STELLARIS 8 FALCON inverted microscope used in this study does not support simultaneous scanning at different laser wavelengths. This posed a particular challenge in analyzing HSPCs, which are highly mobile suspension cell types, potentially leading to registration errors in sequential scans. As a compromise, we collected NAD(P)H and FAD signals in a single scan at the same excitation wavelength (740 nm in the two-photon mode) to enable pixel-level analysis. Such technical compromise will likely result in some NAD(P)H signal saturating into the FAD collection wavelength. Mathematically, we assumed that the collected FAD signal consists of true FAD signal with some “contaminated” signal from NAD(P)H, and the contamination can be estimated as a fraction (α, a constant) of the NAD(P)H signal. Therefore, the observed ORR is a sum of the true ORR and the constant α, as shown in the derivation below

$$\begin{array}{cc} {\text{ORR}}_{\text{observed}}= & \frac{{\text{FAD}}_{\text{observed}}}{\text{NAD}\left(P\right)H}=\frac{{\text{FAD}}_{\text{true}}+\alpha \times \text{NAD}\left(P\right)H}{\text{NAD}\left(P\right)H}=\\ & {\text{ORR}}_{\text{true}}+\alpha \end{array}$$

To focus on the mitochondria metabolism, ORR was calculated as the FAD/NAD(P)H intensity ratio in the mitochondria region and corrected by subtracting the intensity ratio in the cytoplasmic/nuclear region (as background baseline α).

### Live-cell mitochondria staining and imaging

Freshly isolated HSCs (Hoxb5^+^ cKit^+^Lin-Sca^+^), MPPs (Hoxb5^−^ cKit^+^Lin-Sca^+^), and CPs (cKit^+^Lin-Sca^−^) from Hoxb5–tri-mCherry mice were incubated with 50 μM Verapamil (Sigma-Aldrich, V4629) and 100 nM Mitotracker Deep Red FM (Thermo Fisher Scientific, M22426) for 30 min. Cells were then washed with PBS and immobilized with CyGEL Sustain (Abcam, ab109205). Mitochondria and FAD were subsequently imaged under normal confocal mode (excitation: 640 nm, emission: 650/680 nm) and FLIM mode, respectively. The acquired channels were then analyzed to determine the colocalization of mitochondria and FAD (fig. S4).

### TMRM and 6NBDG uptake assay

HSCs were harvested and seeded on the anti-CD43 (eBioR2/60, eBioscience) micropatterned petridish. After 48 hours of culture, cells were first imaged under FLIM mode using a Leica STELLARIS 8 FALCON inverted microscope with each cell’s location recorded. For TMRM staining, cells were incubated with 20 nM TMRM for 30 min and imaged in standard z-stack confocal mode subsequently under 561 nm excitation and 600/40 nm emission. For 6NBDG staining, cells were starved in glucose-free Dulbecco’s modified Eagle medium for 30 min and then incubated with 6NBDG for 30 min and imaged in standard z-stack confocal mode subsequently under 488-nm excitation and 515/20-nm emission. Throughout the imaging process, cells were maintained in a live-cell workstation set at 37°C with 5% CO_2_. Cellular 6NBDG uptake was calculated as the integrated fluorescent intensity over all z-stack slices after subtracting the background. TMRM mean fluorescence intensity was calculated as the integrated fluorescent intensity normalized by the TMRM-positive regions after thresholding. FLIM profile and TMRM/6NBDG signals were then compared between PDCs or at the population level.

### Single cell and subcellular MOB feature extraction

We collected FAD and NAD(P)H fluorescence intensities, as well as the phase and modulation information of NAD(P)H fluorescence lifetime. α_bound_ and τ_bound_ of NAD(P)H were then derived at the pixel level using the previously established phasor approach (*20*). Briefly, the acquired raw information was first converted to the two-dimension phasor plot (commonly noted as the g and s coordinates) at the pixel level. In the analysis, a 3 by 3 median filter was applied to both the g-coordinate and s-coordinate matrices of the image to reduce the noise in fluorescence lifetime measurements. We typically achieved an average of 20 to 30 photons per pixel in our live-cell imaging and analysis. Under the phasor plot, a universal semicircle represents the fluorescence lifetime values from 0 to infinite (*19*). The lifetime of simple components, like enzyme-bound or free NAD(P)H, is located on the semicircle. We have previously determined that the lifetime of free NAD(P)H of hematopoietic cells is close to a constant (i.e., τ_free_ = 0.45 ns), while the lifetime of enzyme-bound NAD(P)H is variable, depending on the enzyme (*20*). Since in each pixel of the cell images there is usually a mixture of both enzyme-bound and free NAD(P)H, the position of the pixels would locate inside the semicircle in between enzyme-bound and free NAD(P)H. By fixing the location of free NAD(P)H, we can calculate the lifetime of enzyme-bound NAD(P)H (τ_bound_) by connecting the g-s coordinates of each pixel with the τ_free_ coordinates and extending the line to intersect with the semicircle. α_bound_ was calculated as the ratio of the distance of the pixel g-s coordinates to τ_free_ over the total length of the line between τ_bound_ and τ_free_ on the phasor plot. Individual cells were then masked from the background based on the NAD(P)H intensity and segmented by a customized Python code (Python 3.9). The intensity threshold for this was set to distinguish the relatively brighter cell signals from the low photon–count background. Typically, the threshold was 1 or 2 photon counts per pixel, determined by our use of low excitation laser power in live-cell imaging, which generally results in minimal background photon counts. Following cell identification, we generated a cell mask for the entire image. To segment individual cells, we applied the binary_erosion function from the scipy.ndimage.morphology library to erode the mask. Subsequently, we used the watershed algorithm from skimage.segmentation to further refine single-cell segmentation. During this process, size-based exclusion criteria were implemented to eliminate debris and unsegregated cell clumps. After background subtraction, pixel-level FAD intensity in individual cells was used to segment the mitochondria and cytoplasmic/nuclear regions by Otsu’s thresholding method (figs. S3 and S4). In both regions of a single cell, NAD(P)H intensity, α_bound_, τ_bound_, and ORR (only in the mitochondria region) were respectively quantified using a collection of defined features describing the signal strength (average, median, 10 percentile, 90 percentile, etc.), statistical distribution (SD, skewness, kurtosis, etc.), and spatial variance/texture [difference variance of gray level co-occurrence matrix, gray level variance of gray level run length matrix, etc. (*75*); see data S1]. Compartmentalization of individual features between the two regions was also quantified as

$$\text{compartmentalization}=\frac{{\text{feature}}_{\text{mitochondria}}-{\text{feature}}_{\text{cytoplasm}}}{{\text{feature}}_{\text{mitochondria}}+{\text{feature}}_{\text{cytoplasm}}}$$

A set of morphological features were also developed using mostly the whole cell masks (size, major axis length, circularity, etc.) and/or mitochondria (size and dispersity) and cytoplasm/nucleus (size). Extraction of all above features was processed using a customized Python code with image processing packages, including SciPy, Scikit-image, PyRadiomics, and SimpleITK.

### MOB feature selection

“Informative” MOB features were selected on the basis of the prediction performance (in a defined machine learning model) and the monotony of the trending in both in vitro differentiation and HSPC hierarchy datasets. For each individual feature, a logistic regression model was trained with 60% of the sample data, and the F1 score was used to evaluate the prediction performance using the rest 40% of data. The F1 score is defined as

$$F1\;\text{score}=\frac{2\left(\text{precision}\times \text{recall}\right)}{\text{precision}+\text{recall}}$$

where the precision is the ratio of correctly predicted HSCs in all predicted HSCs, and recall is the ratio of correctly predicted HSCs in all HSCs. The threshold of the F1 score was set to 0.6. Features scored below this value were considered as not able to distinguish HSC and differentiated cells and thus dropped. The monotony of individual features were quantified by the trending index (TI) during the differentiation, defined as

$$\text{TI}=\frac{\sum_{t_{n}=1}\left[\text{feature}\left(t_{n}\right)-\text{feature}\left(t_{n-1}\right)\right]}{\sum_{t_{n}=1}|\text{feature}\left(t_{n}\right)-\text{feature}\left(t_{n-1}\right)|}$$

where the feature(*t_n_*) represents the population level average of an individual feature at *t_n_*, and *t_n_* refers to the differentiation stages of HSC in specific datasets. In HSPC hierarchy, *t_n_* = 0 for HSCs, *t_n_* = 1 for MPPs, and *t_n_* = 2 for CPs; in HSC in vitro culture, *t_n_* = 0 for freshly isolated HSCs, *t_n_* = 1 for cultured HSCs on day 2, *t_n_* = 2 for day 4, and *t_n_* = 3 for day 7. If the feature changes in a monotonous trend during HSC differentiation, then TI equals or is close to 1 (continuously increasing) or −1 (continuously decreasing). Features with TI in between −0.5 and 0.5 were considered as “fluctuating” (not monotonous) and thus dropped. Eventually, we examined each feature and only selected those meet the following two criteria: (i) F1 score > 0.6 in both datasets and (ii) TI below −0.5 or above 0.5 in both datasets.

On the basis of the above criteria, 56 MOB features were selected of 205. Next, a knowledge graph reflecting the relationship of different features was plotted to select the representative features based on the enrichment of the 56 features in subcategories. The knowledge graph contained four stem edges: morphology, mitochondria, cytoplasm/nucleus, and compartmentalization. Under each stem edge, branch edges and nodes were plotted to represent information in the specific region/ category. For example, the average, median, 10 percentile, and 90 percentile of NAD(P)H intensity in the mitochondria region all reflect the signal strength of NAD(P)H in mitochondria and are correlated. Since they all pass the feature selection process, it indicates that NAD(P)H intensity in mitochondria is informative in profiling HSC differentiation, and thus the average NAD(P)H intensity in mitochondria was selected as a representative feature to reflect this fact. In contrast, in the node of ORR statistical distribution in mitochondria, only kurtosis passed the feature selection, while entropy, interquartile range, skewness, uniformity, and variance did not pass, indicating that ORR statistical distribution is less informative, and this node was thus dropped.

### Data trimming and normalization

Upon MOB feature extraction for single cells, outliers were removed using a density-based spatial clustering of applications with noise algorithm in the Scikit-learn package. Cells within each population or experimental condition were shuffled using the shuffle algorithm in the Scikit-learn package and then trimmed to the same number to avoid bias in the analysis.

Datasets collected from independent experiments were normalized with a Robust Scaler from Python machine learning package Scikit-learn, using HSCs (for extended in vitro culture and HSPC hierarchy) or all cells (for PDC studies) as the training set. The Robust Scaler adjusts the population median to zero and rescales the data according to the range between the first quartile (25% quartile) and the third quartile (75% quartile), such that the median is rescaled to 0, the first quartile is rescaled to −1, and the third quartile is rescaled to 1.

### Trajectory inference and pseudotime analysis

In the HSPC and extended in vitro culture datasets, the 56 selected features were normalized, and trajectory inference was performed using algorithms from ScanPy package with a customized Python code (*23*). The dimension of the datasets was reduced to the first eight principal components, the Louvain method was used for community detection, and partition-based graph abstraction was used to infer the differentiation path. Pseudotime was then calculated using the diffusion pseudotime algorithm in the package.

### ROC curve and latent variable model

The ROC curve was used to evaluate the HSC-differentiated cell prediction accuracy from the selected MOB features. In each dataset, an SVM model was trained with 70% of the single-cell data using all the 205 extracted features, the 11 selected features, or a single feature (cell size). The true-positive rate, false-positive rate, and area under the curve were calculated and plotted with the rest 30% single-cell data.

For the latent variable analysis, the 11 selected MOB features were fitted into a regression model with a customized Python code calling the factor analysis algorithm in the Scikit-learn package. The dimensionality of the latent space was set as 3, and the initial guess of the noise variance for each feature in the model was set as 0. The primary latent variable (LV1) was defined as the MOB score. The correlation coefficients between the primary latent variable and the MOB features were considered as the weight of each feature.

### Time-lapse tracking of HSC division

The micropatterned single HSCs were tracked for the division under a Leica STELLARIS 8 FALCON inverted microscope with a live-cell workstation (37°C, 5% CO_2_). To quantify the correlation of MOB profile between parent and daughter cells, single HSCs were imaged 12 hours after seeding with their location recorded. Forty-four hours after seeding, daughter cells on the same location were imaged and corresponded to the original parent HSCs. For time-lapse imaging of the metabolic changes before and after division, freshly isolated HSCs were seeded, incubated at 37°C, 5% CO_2_ for 24 hours, and then imaged under a Zeiss LSM-780 inverted microscope coupled with an A320 FastFLIM FLIMbox (ISS Inc.) at 30-min intervals for 24 hours. For tracking the fate of daughter cells from different division patterns, PDCs were first imaged 40 hours after the initial parent HSC seeding with their location recorded. After another 8 hours, these cells were imaged again, and the results were compared.

### Immunostaining of surface markers in PDCs

Paired cells on the micropatterned antibody array were first imaged under FLIM mode using a Leica STELLARIS 8 FALCON inverted microscope with each cell’s location recorded. Cells were then washed with PBS and fixed with 4% paraformaldehyde (Electron Microscopy Sciences) in PBS at room temperature for 12 min. After washing with PBS, cells were blocked with 4% HyClone bovine serum albumin (BSA; Cytiva) in PBS at room temperature for 2 hours. Cells were then stained with Alexa Fluor 647–conjugated Tie2 antibody (clone 33, BD Biosciences; 1:100 dilution in 4% BSA) and phycoerythrin-conjugated CD48 antibody (HM48-1, BioLegend; 1:100 dilution in 4% BSA) at room temperature for 2 hours (*26*). Imaging of surface markers was carried out on the same microscope under the normal confocal mode.

### HSC division pattern analysis after metabolic intervention

Freshly isolated HSCs were seeded on the abovementioned customized petridishes with anti-CD43 micropatterns on the #1 glass bottom, with single HSCs trapped on individual micropatterns. These HSCs were cultured in the absence or presence of reagents that regulate metabolism (table S4) for 24 hours. The reagent-containing medium was then removed, and the HSCs were washed two times with PBS. Cells were then cultured with standard medium for another 20 hours to allow cell division. The MOB profile of PDCs was acquired for division pattern analysis.

### HSC culture with PI3K inhibitors and CFU assay

Freshly isolated HSCs were seeded into a 96-well plate (Corning) with the initial density of 200 cells per well and cultured in the absence or presence of 5 μM LY294002 or 5 nM copanlisib for 7 days, with half-medium changes on day 4. On day 7, cells were harvested, washed with 2% fetal bovine serum (FBS, Sigma-Aldrich) in PBS, immunostained with HSC surface marker cocktail (table S1) for 1.5 hours, and analyzed by a BD SORP FACSAria cell sorter. For CFU assay, harvested cells were counted with a hemacytometer and then washed with Iscove’s modified Dulbecco’s medium (IMDM) with 2% FBS. Harvested cells (1/100) from each well (equivalent to cells expanded from two initial HSCs) were resuspended in 100 μl of IMDM with 2% FBS and then mixed with 1 ml of MethoCult GF M3434 medium (STEMCELL Technologies), seeded into a six-well clear-bottom plate (Corning), and incubated at 37°C with 5% CO_2_. After 7 and 10 days of culture, colonies from each well were imaged under a Nikon Inverted Microscope Eclipse Ti-E with a 2× objective. Different colony types, including CFU–granulocyte/macrophage/granulocyte and macrophage (colorless, consisted of round cells and/or larger oval cells), burst-forming unit–erythroid (usually reddish, consisted of small, irregular shaped cells that appear fused together), and CFU–granulocyte, erythrocyte, macrophage, megakaryocyte (a single colony that shows a mixture of cell types from the first two colonies), were manually counted.

### Data plot and statistical analysis

All plots were made in Prism 7 (GraphPad) and Python 3.9 (Python Software Foundation). For analysis between PDCs, statistical analysis was performed with paired *t* test. Otherwise, statistical analysis for single-cell scatter or box plots was performed with the Mann-Whitney (2 conditions) or Kruskal-Wallis tests (>2 conditions) due to nonnormal data distribution determined by the normality test, except otherwise indicated. All bars in the scatterplot are median, and box plots represent the 10th to 90th percentiles. All error bars in the bar graphs were plotted as SD and compared with Welch’s *t* test (2 conditions) or ordinary one-way analysis of variation (ANOVA) (>2 conditions). Correlation analysis was carried out with Pearson *r*, and *P* values were tested with zero-slope hypothesis. *P* values are indicated as numbers or as n.s. (not significant, *P* > 0.05) on the plots.
