# Supplementary material for: Label-free metabolic optical biomarkers track stem cell fate transition in real time
Source: Sci Adv. 2024 May 8;10(19):eadi6770. doi: 10.1126/sciadv.adi6770 (PMC11078180; doi:10.1126/sciadv.adi6770)
Supplement: Supplementary file 1 — Figs. S1 to S10 Tables S1 to S4 Legend for data S1 [file sciadv.adi6770_sm.pdf]

Science Advances
AAAS

Supplementary Materials for

Label-free metabolic optical biomarkers track stem cell fate transition in real time

Hao Zhou *et al.*

Corresponding author: Keyue Shen, keyue.shen@usc.edu

Sci. Adv. **10**, eadi6770 (2024)
DOI: 10.1126/sciadv.adi6770

The PDF file includes:

Figs. S1 to S10
Tables S1 to S4
Legend for data S1

Other Supplementary Material for this manuscript includes the following:

Data S1

Supplementary Figures

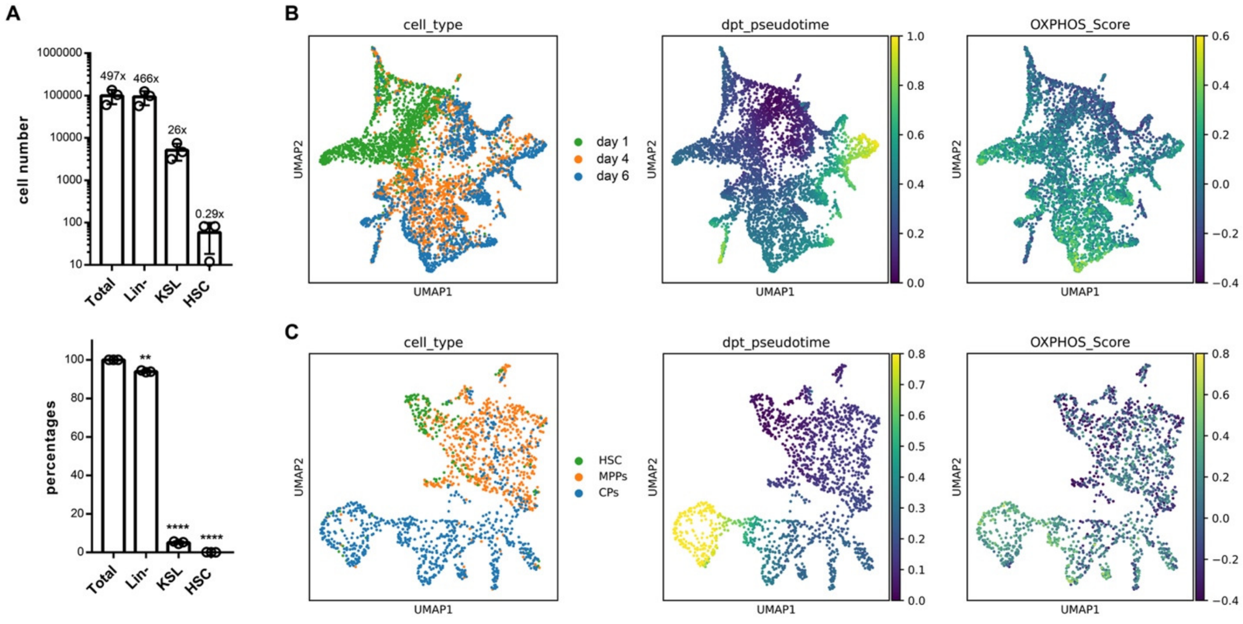

Fig. S1 | Characterization of HSC differentiation.

(A) Phenotypic profiling and quantification of cells expanded *in vitro* from 200 HSCs in one week. n = 3 biological replicates. p-values: one-way ANOVA. **: p<0.01; ****: p<0.0001.

Single-cell RNA-seq based trajectory inference and OXPHOS evaluation of hematopoietic stem and progenitor cells differentiation (B) *in vitro*, analyzed with data from reference (*5*); and (C) *in vivo*, analyzed with data from reference (*49*).

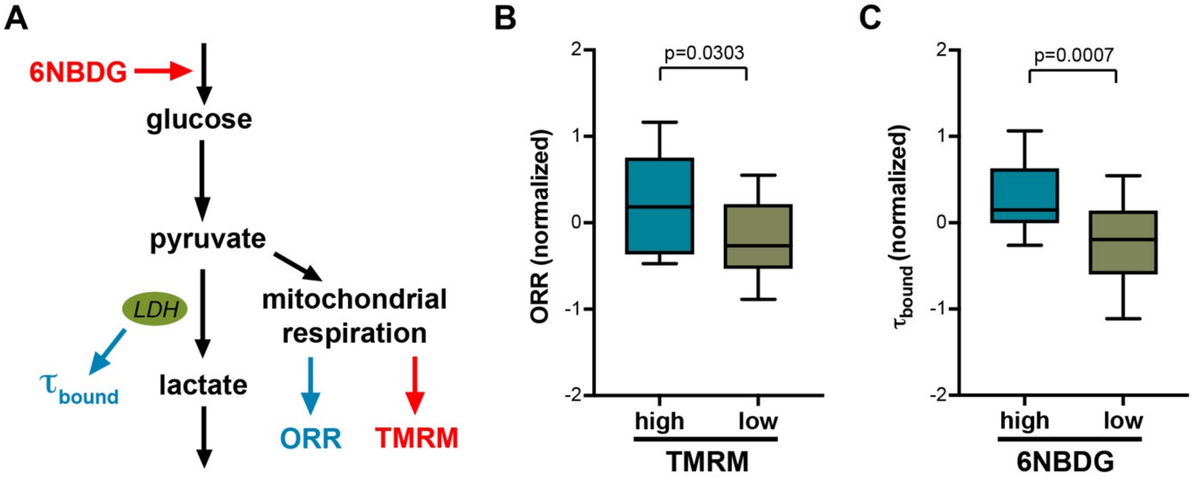

Fig. S2 | Correlation between FLIM features and metabolic dyes.

(A) Illustration of metabolic dyes in profiling OXPHOS and glycolysis.

(B) ORR readout in TMRM-high (top 50%) and TMRM-low (bottom 50%) cells. n = 78 cells. p-values: Mann-Whitney test.

(C) τ_{bound} readout in 6NBDG-high (top 50%)) and 6NBDG-low (bottom 50%)) cells. n = 60 cells. p-values: Mann-Whitney test.

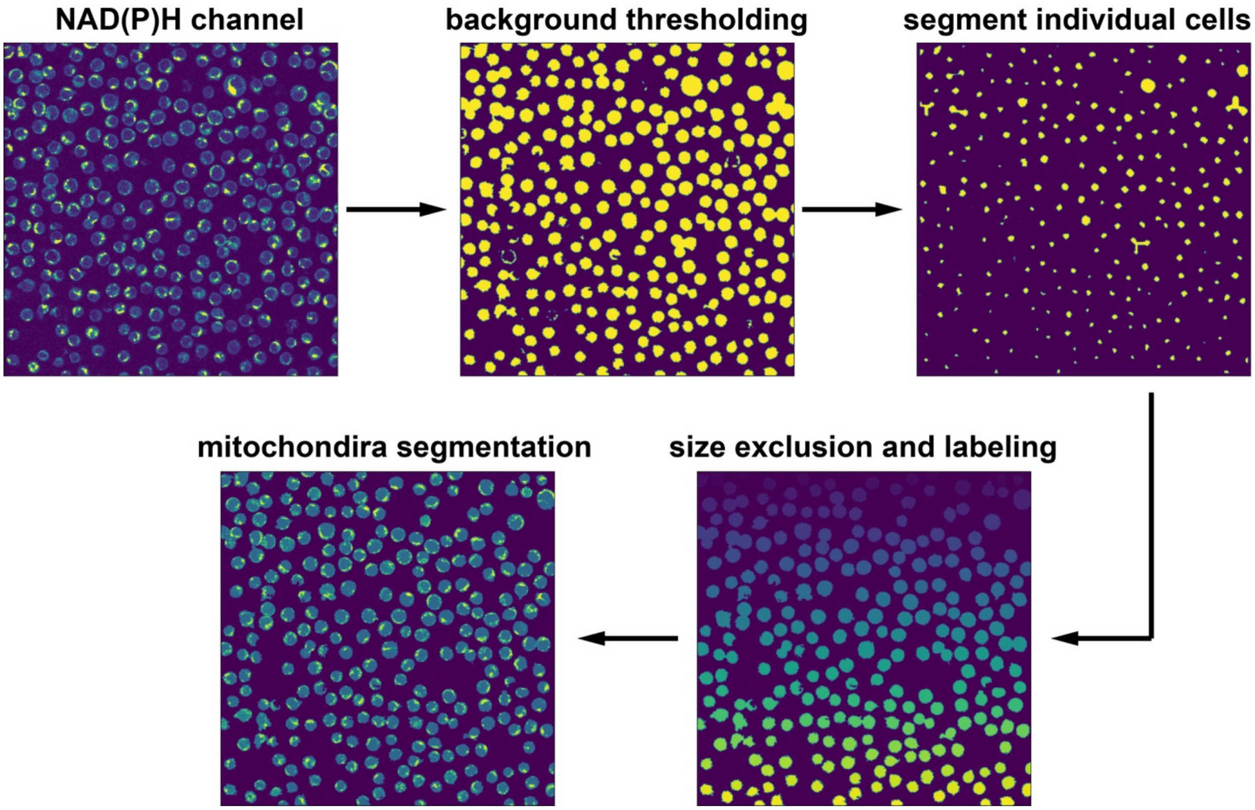

Fig. S3 | The workflow for cell masking, single cell isolation, and mitochondria segmentation.

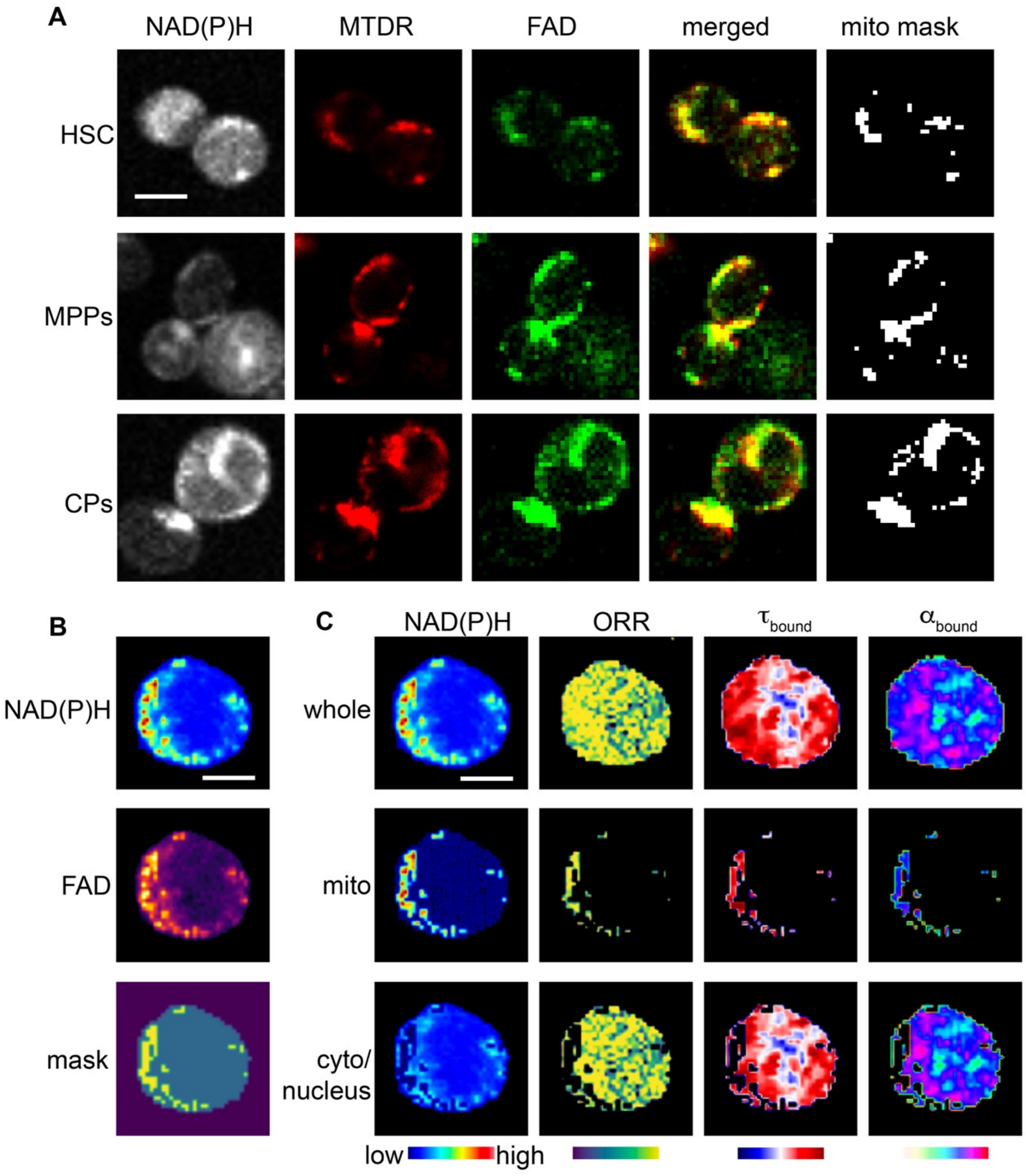

Fig. S4 | Mitochondria segmentation based on FAD intensity.

(A) Colocalization of FAD enriched region with MitoTracker Deep Red in hematopoietic stem and progenitor cells.

(B) An example of mitochondria segmentation based on FAD signal.

(C) An example of separate FLIM analysis in mitochondrial region and cytoplasmic/nuclear region. Scale bars: 5 µm.

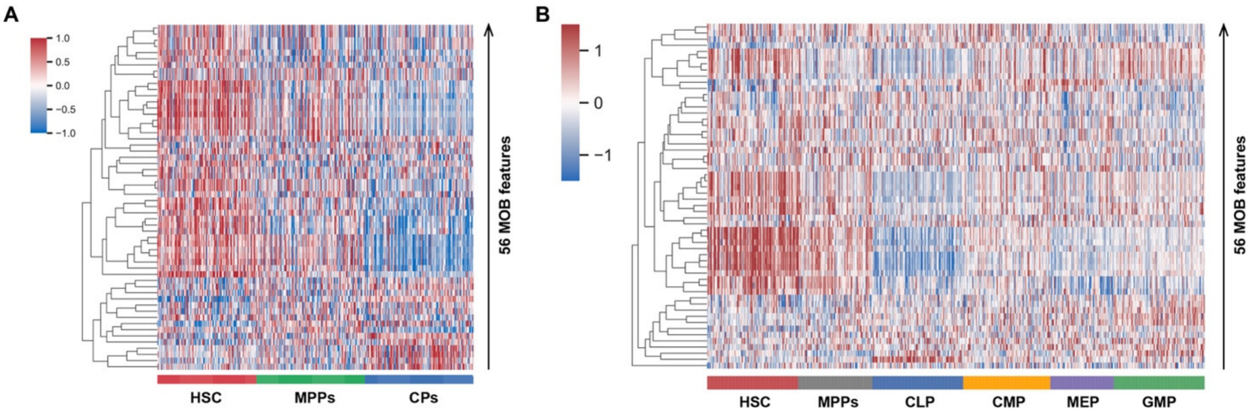

Fig. S5 | FLIM-derived MOB features profile HSC differentiation.

(A) and (B) Heatmap of 56 features passing machine learning-based and monotonous trending-based selection in HSPC hierarchy models (n = 437 cells and n = 548 cells respectively).

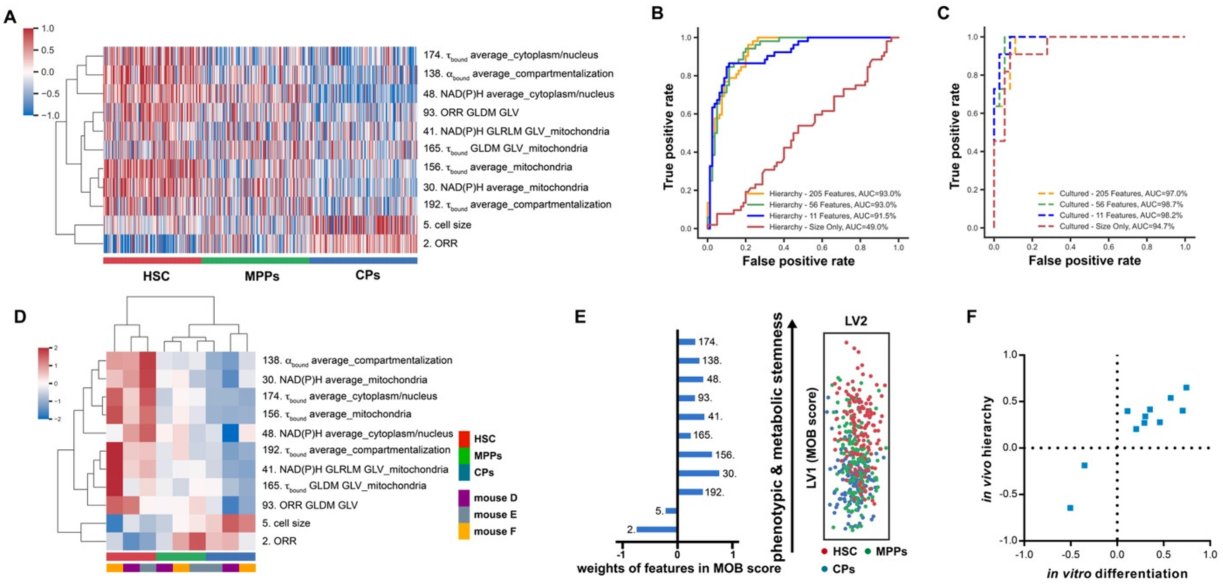

Fig. S6 | MOB score is a robust metric in HSC differentiation.

(A) Heatmap of 11 representative MOB features in HSPC hierarchy (n = 437 cells).

(B) and (C) ROC curves indicating the prediction accuracy by the support vector machine (SVM) models using different features in HSPC hierarchy and HSC *in vitro* differentiation.

(D) Hierarchical clustering showed that MOB features can track HSPC hierarchy in independent experiments.

(E) Latent variable analysis of MOB features and their weights in MOB score.

(F) Correlation between weights of MOB features derived from HSC *in vitro* differentiation and from HSPC hierarchy.

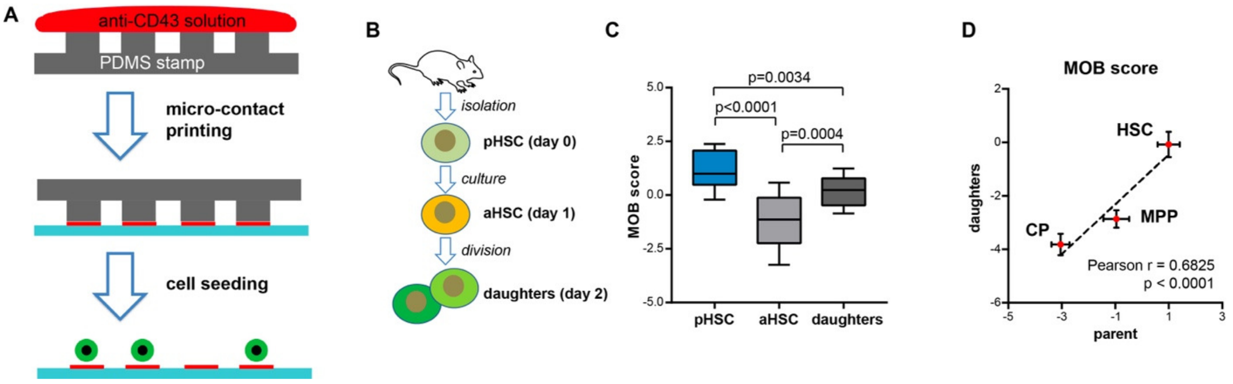

Fig. S7 | MOB score reveals inheritance of metabolic phenotype during HSPC division.

(A) Schematic of trapping single HSCs with micro-contact printed antibody array.

(B) Schematic of HSC metabolic changes during the first cell cycle *in vitro*.

(C) MOB score of HSCs during the first division *in vitro*. pHSC: primary HSC; aHSC: activated HSC after 20 hours of *in vitro* incubation; daughters: daughter cells from the 1st division imaged at 44-hour. n = 38 divisions. p-values: Kruskal-Wallis test.

(D) Correlation of MOB score between parent and daughter cells in different HSPC populations. n = 85 divisions. Error bars: standard deviation.

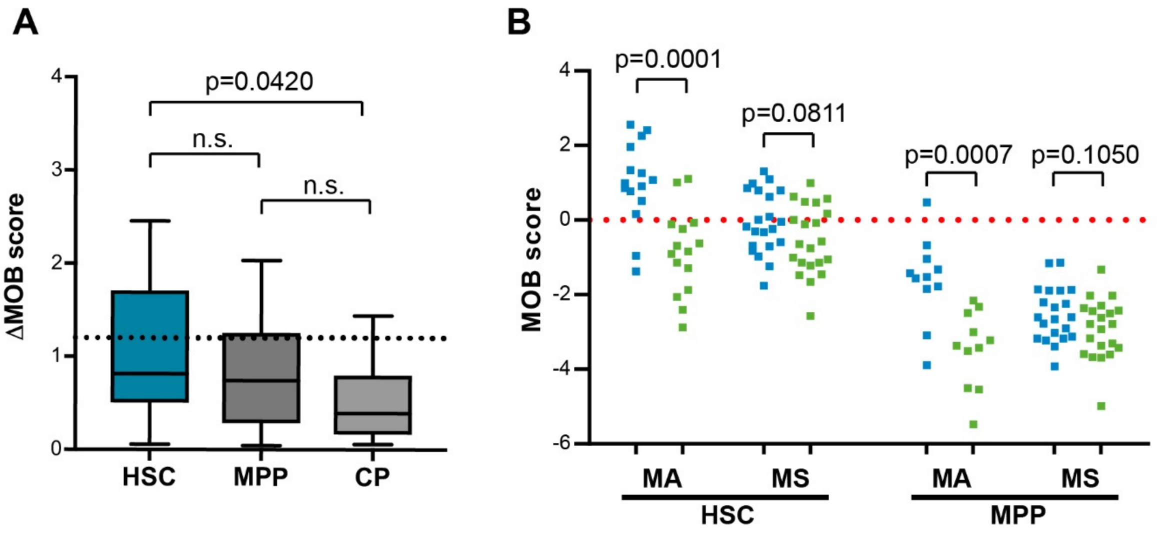

Fig. S8 | MOB score reveals different division patterns of HSPC populations.

(A) Metabolic asymmetry of HSPC division quantified by ΔMOB score. n= 84 cell pairs. p-values: Kruskal-Wallis test.

(B) MOB score of HSC and MPP daughter cells under different division patterns. n= 68 cell pairs. p-values: paired t-test.

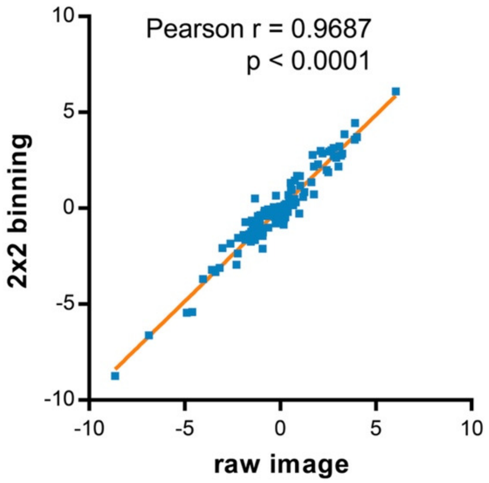

Fig. S9 | MOB scores correlate between regular and high (2×2 binned) photon counts from FLIM images of hematopoietic stem cells. The 2×2 pixel binning on raw images quadruples the photon counts per pixel. Each dot represents the MOB scores of a single hematopoietic stem cell.

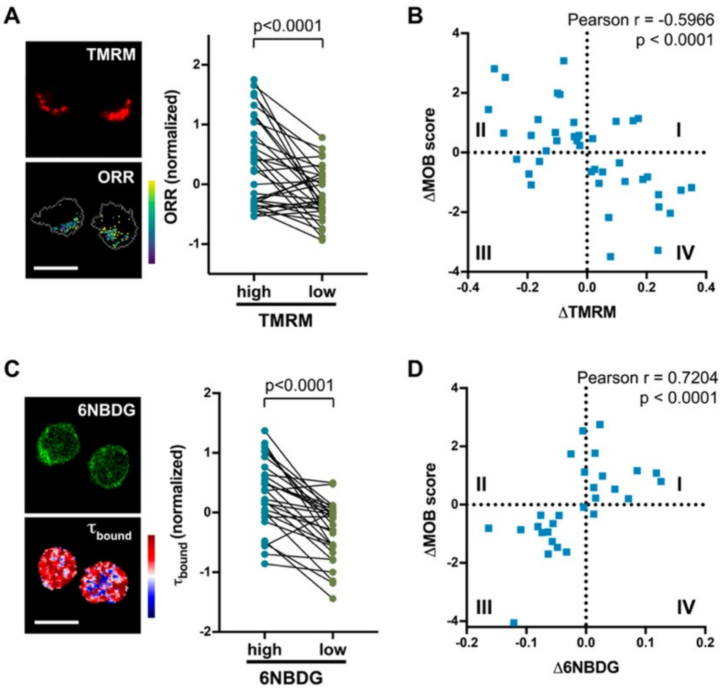

Fig. S10 | MOB features and score identify the metabolic asymmetry in paired daughter cells.

(A) Representative images of differential TMRM uptake in PDCs and the corresponding ORR. Daughter cells exhibiting higher TMRM intensity also demonstrate significantly elevated ORR values compared to their counterparts with lower TMRM signal. Scale bar: 10 µm. n = 33 cell pairs (dots connected by lines). p-values: paired t-test.

(B) A significant negative correlation exists between ΔMOB score and ΔTMRM intensity in PDCs, and the two values have the opposite signs in most cell pairs.

(C) Representative images of differential 6NBDG uptake in PDCs and the corresponding τ_{bound}. Scale bar: 10 µm. n = 29 cell pairs. p-values: paired t-test.

(D) A significant positive correlation exists between ΔMOB score and Δ6NBDG uptake in PDCs, and the two values have the same signs in most cell pairs.

Supplementary Tables

Table S1. *Surface antigens used for sorting hematopoietic stem and progenitor cell populations.*

Cell type	Surface antigens
HSC	*Lin- cKit+ Sca1+ Flk2- CD34- Slamf1+
MPP	Lin- cKit+ Sca1+
CP	Lin- cKit+ Sca1-
MPP^{Flk2-}	Lin- cKit+ Sca1+ Flk2- CD34+
MPP^{Flk2+}	Lin- cKit+ Sca1+ Flk2+
CLP	Lin- IL7rα+ Flk2+
CMP	Lin- cKit+ Sca1- FcγR- CD34+
MEP	Lin- cKit+ Sca1- FcγR- CD34-
GMP	Lin- cKit+ Sca1- FcγR+
Lin-CD45+	Lin- CD45.2+
CD45+	CD45.2+
	*Lin (Lineage) markers include B220, CD3, CD4, CD8, Gr1, Mac1 and Ter119.

Table S2. *Antibodies used for sorting.*

Antigen	Conjugation	Vendor	Catalog#	Clone
B220	PerCP-Cy5.5	ebioscience	45-0452-82	6B2
CD3	PerCP-Cy5.5	ebioscience	45-0031-82	KT31.1
CD34	e660	ebioscience	50-0341-82	RAM34
CD4	PerCP-Cy5.5	ebioscience	45-0042-82	GK1.5
CD8	PerCP-Cy5.5	ebioscience	45-0081-82	53-6.7
cKit	APC-EF780	ebioscience	47-1171-82	2B8
FcγR	Biotin	ebioscience	13-0161-85	93
Flk2	PE-Cy5	ebioscience	15-1351-81	A2F-10
Gr1	PerCP-Cy5.5	Affymetrix	45-5931-80	8C5
IL7rα	PE-Cy7	Biolegend	135013	A7R34
CD45.2	A700	Biolegend	109822	104
CD45.1	PE-eFluor 610	Affymetrix	61-0453-82	A20
Mac1	PerCP-Cy5.5	ebioscience	45-0112-82	M1/70
Sca1	BV711	Biolegend	108131	D7
Slamf1	PE	Biolegend	115904	TC15-12F12.2
Biotin	PE-Cy5.5	Life technologies	SA1018	
Ter119	PerCP-Cy5.5	ebioscience	45-5921-82	Ter119

Table S3. *11 representative MOB features used for scoring.*

log number	feature name	abbreviation or alias for figure plot	definition	implication
2	ORR_FAD/NADH	ORR	mitochondrial optical redox ratio (ORR=FAD/NAD(P)H) subtracts the baseline in cytoplasm/nucleus	mitochondrial redox state and OXPHOS
5	size_whole	cell size	area of cell in the image	cell size
30	i_NADH-mito-original_firstorder_Mean	NAD(P)H average_mitochondria	average NAD(P)H intensity in the mitochondrial region	mitochondrial NAD(P)H concentration
41	i_NADH-mito-original_glrlm_GrayLevelVariance	NAD(P)H GLRLM GLV_mitochondria	GLRLM-gray level variance of NAD(P)H intensity in the mitochondrial region	mitochondrial NAD(P)H spatial variance
48	i_NADH-cyto-original_firstorder_Mean	NAD(P)H average_cytoplasm	average NAD(P)H intensity in the cytoplasmic/nuclear region	cytoplasmic/nuclear NAD(P)H concentration
93	ORR-mito-original_gldm_GrayLevelVariance	ORR GLDM GLV	GLDM-gray level variance of ORR in the mitochondrial region	mitochondrial redox state and OXPHOS spatial variance
138	abound-compartment-original_firstorder_Mean	abound average_compartmentalization	difference of average α_{bound} between mitochondrial and cytoplasmic/nuclear regions	NAD(P)H-protein binding compartmentalization between mitochondrial and cytoplasmic/nuclear region

156	tbound-mito-original_firstorder_Mean	tbound average_mitochondria	average τ_{bound} in the mitochondrial region	glycolysis in the mitochondrial region
165	tbound-mito-original_gldm_GrayLevelVariance	tbound GLDM GLV_mitochondria	GLDM-gray level variance of τ_{bound} in the mitochondrial region	spatial variance of glycolysis in the mitochondrial region
174	tbound-cyto-original_firstorder_Mean	tbound average_cytoplasm	average τ_{bound} in the cytoplasmic/nuclear region	glycolysis in cytoplasmic/nuclear region
192	tbound-compartment-original_firstorder_Mean	tbound average_compartmentalization	difference of average τ_{bound} between mitochondrial and cytoplasmic/nuclear regions	glycolysis compartmentalization between mitochondrial and cytoplasmic/nuclear region

Table S4. *Reagents used for metabolic intervention.*

Reagent	Vendor	Catalog#	Work Concentration	Function
PS48	Sigma-Aldrich	P0022	5 µM	Phosphoinositide-dependent protein kinase 1 activator
Sodium oxamate	Cayman Chemical	19057	10 mM	Lactate dehydrogenase inhibitor
Rapamycin	Sigma-Aldrich	553210	1 µM	mTOR inhibitor
LY294002	MedChemExpress	HY-10108	5 µM	Pan PI3k inhibitor
Copanlisib	MedChemExpress	HY-15346	5 nM	Pan PI3k inhibitor
N-Acetyl Cysteine	Millipore Sigma	A7250	100 µM	Reductant
Tert-Butyl hydroperoxide	Millipore Sigma	458139	100 µM	Oxidant

Data S1. (separate file)

A full list of the 205 MOB features used in data analysis.
